# Guanidinylated Polymyxins as Outer Membrane Permeabilizers Capable of Potentiating Rifampicin, Erythromycin, Ceftazidime and Aztreonam against Gram-Negative Bacteria

**DOI:** 10.3390/antibiotics11101277

**Published:** 2022-09-20

**Authors:** Danzel Marie Ramirez, Danyel Ramirez, Gilbert Arthur, George Zhanel, Frank Schweizer

**Affiliations:** 1 Department of Chemistry, University of Manitoba, Winnipeg, MB R3T 2N2, Canada; 2 Department of Biochemistry and Medical Genetics, University of Manitoba, Winnipeg, MB R3E 0W2, Canada; 3 Department of Medical Microbiology and Infectious Diseases, University of Manitoba, Winnipeg, MB R3E 0J9, Canada

**Keywords:** outer membrane permeabilizer, antibiotic potentiator, antibiotic adjuvant, polymyxins, *Pseudomonas aeruginosa*, *Acinetobacter baumannii*, *Enterobacteriaceae*

## Abstract

Polymyxins are considered a last-line treatment against infections caused by multidrug-resistant (MDR) Gram-negative bacteria. In addition to their use as a potent antibiotic, polymyxins have also been utilized as outer membrane (OM) permeabilizers, capable of augmenting the activity of a partner antibiotic. Several polymyxin derivatives have been developed accordingly, with the objective of mitigating associated nephrotoxicity. The conversion of polymyxins to guanidinylated derivatives, whereby the L-γ-diaminobutyric acid (Dab) amines are substituted with guanidines, are described herein. The resulting guanidinylated colistin and polymyxin B (PMB) exhibited reduced antibacterial activity but preserved OM permeabilizing properties that allowed potentiation of several antibiotic classes. Rifampicin, erythromycin, ceftazidime and aztreonam were particularly potentiated against clinically relevant MDR Gram-negative bacteria. The potentiating effects of guanidinylated polymyxins with ceftazidime or aztreonam were further enhanced by adding the β-lactamase inhibitor avibactam.

## 1. Introduction

The rising prevalence of antibiotic resistance in pathogenic bacteria, coupled with the decline in research and development of novel antibiotics due to perceived poor return on investment and scientific limitations, raises a global health concern [1,2]. An advantageous approach to overcome antibiotic resistance while repurposing current antibacterial agents is through the utilization of adjuvants or potentiators [3]. Adjuvants can resuscitate the activity of antibiotics against resistant strains and expand the activity spectrum of antibiotics [3]. In addition, the synergistic interaction between the adjuvant and antibiotic results in enhanced activity with a reduction in the concentration of both agents. This allows antibiotics to be efficacious at lower doses and potentially mitigate toxic effects [3]. Furthermore, bacteria are less likely to develop resistance from evolutionary pressure to adjuvants since these molecules generally do not exert bactericidal or growth inhibitory effects [3].

Outer membrane (OM) permeabilizers are a class of adjuvants that interact with and disrupt the integrity of the OM of Gram-negative bacteria [4,5,6]. Increased OM permeability allows the transit of antibiotics that are otherwise restricted, resulting in an increase in the periplasmic and/or intracellular concentration of antibiotics [4,5,6]. Current design strategies for OM permeabilizers focus on the development of polycationic amphiphiles derived from peptides, peptidomimetics, lipopeptides and aminoglycosides [7,8,9,10,11]. Polycationic amphiphiles in the form of polymyxins are some of the most effective known OM permeabilizers [12,13]. Polymyxins are cyclic lipopeptide antibiotics capable of crossing the OM through a mechanism of self-promoted uptake [14]. The mechanism involves electrostatic interactions between the positively charged L-γ-diaminobutyric acid (Dab) residues of polymyxins and the negatively charged lipid A phosphates within the lipopolysaccharide leaflet of the OM [15]. This causes the displacement of divalent magnesium and calcium cations that initially bridge the phosphate groups and provide stability to the lipid components of the membrane [15]. Thus, the removal of these cations results in a transient disruption of the OM and enhanced permeability [15]. The *N*-terminal fatty acyl chain and hydrophobic domains of polymyxins also contribute to OM expansion by inserting and destabilizing the packing of the lipid A fatty acyl layer [15]. The design of existing polymyxin-based OM permeabilizers, such as polymyxin B nonapeptide (PMBN) and SPR741, emphasized alleviating nephrotoxicity [16,17,18,19,20,21] through the removal of the *N*-terminal lipid tail and reduction in the number of Dab residues, leading to a decreased overall positive charge. These two structural changes also result in both analogs lacking potent antibacterial activity. Nonetheless, both analogs retain the ability to bind to the LPS, disrupt the OM and synergize with different antibiotics [12].

In order to design novel polymyxin-based OM permeabilizers, several key factors were taken into consideration. First, the structural modifications should reduce or abolish the antibacterial activity but enhance or retain OM permeabilization. Second, the synthesis should be directly accessible from polymyxins and utilize a low number of synthetic steps with high yields. Lastly, the associated nephrotoxicity and neurotoxicity with the use of polymyxins should also be minimized. Since structure–activity relationship (SAR) studies have established the importance of maintaining the Dab residues [15], the conversion of the amine group within these side chains to a guanidine function was presumed to reduce antibacterial activity. Moreover, this substitution was envisioned to enhance the OM permeabilizing property. In comparison to primary amines, guanidinium groups have a higher p*K*_a_ and remain protonated over a wide pH range [22]. The delocalization of the positive charge and planar Y-shape geometry allow them to bind with high affinity to oxoanions [22], such as the phosphates on the core sugars and lipid A of the LPS [23]. In addition, a study on cationic peptide-based adjuvants has shown increased potentiation by peptides with arginine residues in comparison to lysine and Dab [9].

This study demonstrates that guanidinylation transforms polymyxins into effective OM permeabilizers capable of potentiating multiple classes of antibiotics against clinically relevant multidrug-resistant (MDR) Gram-negative pathogens. Notably, GCol and GPMB synergized with the highly hydrophobic rifampicin and erythromycin, as well as beta-lactams, ceftazidime and aztreonam. In the presence of the guanidinylated polymyxins, the interpretative susceptibility breakpoints for rifampicin and erythromycin were reached against several MDR strains. Furthermore, the synergy of the guanidinylated polymyxins with ceftazidime and aztreonam were further improved upon the addition of a third component, avibactam, against *Pseudomonas aeruginosa* isolates harboring β-lactamases.

## 2. Results

### 2.1. Synthesis of Guanidinylated Polymyxins 

**Figure 1:**
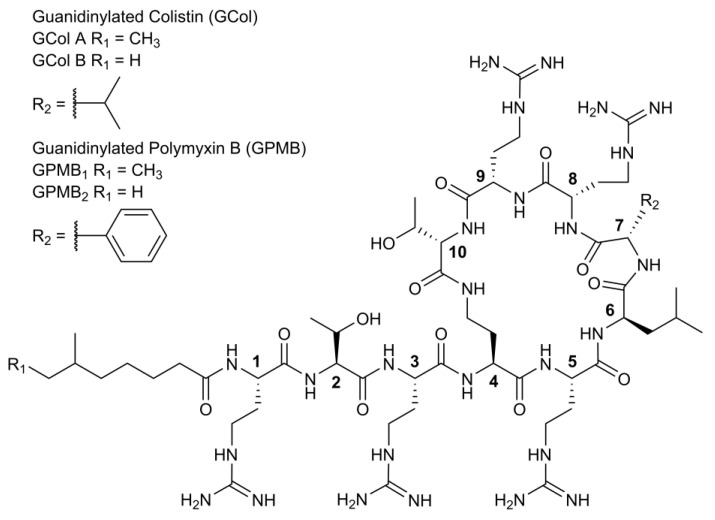
Structure of guanidinylated polymyxins.

The guanidinylation of colistin and polymyxin B (PMB) was carried out using *N,N′*-Bis(tert-butoxycarboyl)-*N″*-trifylguanidine following an established method [24] to yield the guanidinylated polymyxins GCol and GPMB (Figure 1). GCol consists of a mixture of guanidinylated colistin A (58%) and B (42%), while GPMB comprises of a mixture of guanidinylated PMB_1_ (88%) and PMB_2_ (12%). 

### 2.2. Antibacterial Activity of GCol and GPMB against Wild-Type Gram-Negative Bacteria

To ascertain whether the antibacterial action of the guanidinylated polymyxins is lost or retained, the antimicrobial susceptibility assay was evaluated against wild-type Gram-negative bacteria. The guanidinylated polymyxins demonstrated higher minimum inhibitory concentrations (MICs) than the respective parent compounds against *P. aeruginosa* PAO1, *Acinetobacter baumannii* ATCC 17978 and *Escherichia coli* ATCC 25922 (Table 1). For instance, GCol displayed a >128-fold reduction in antibacterial activity in comparison to colistin against the three organisms. Conversely, GPMB displayed an 8-, 16- and 64-fold reduction in antibacterial activity against *E. coli* ATCC 25922, *A. baumannii* ATCC 17978 and *P. aeruginosa* PAO1, respectively when compared to PMB.

### 2.3. Synergy with Different Antibiotics against Wild-Type Gram-Negative Bacteria

**Figure 2:**
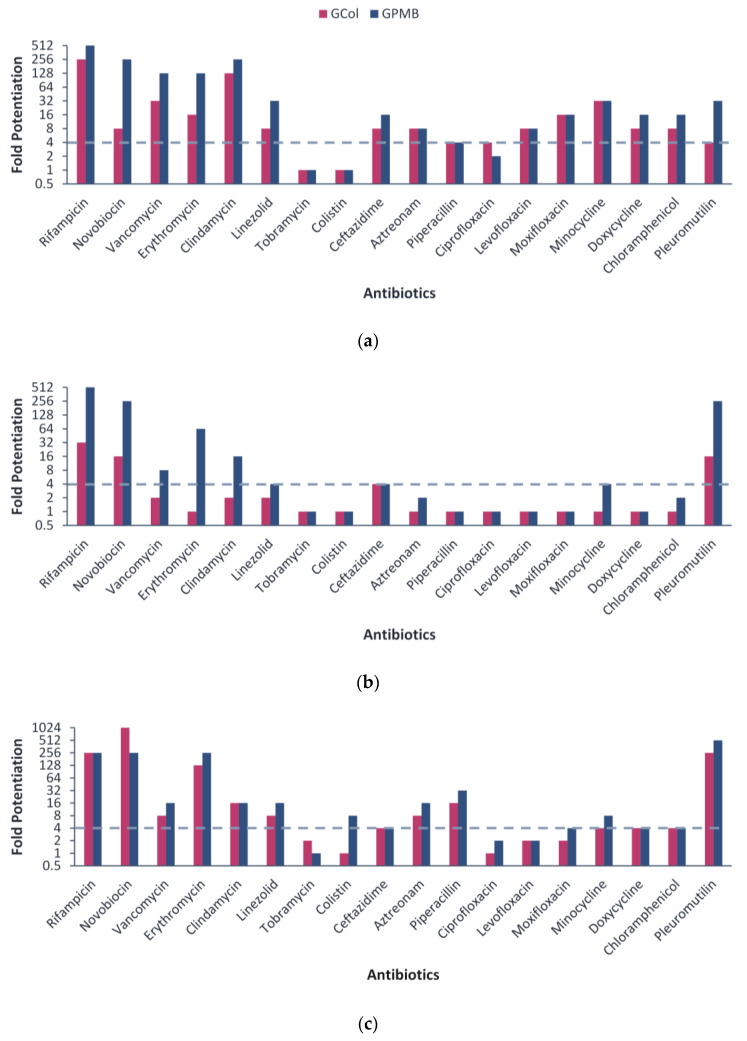
Potentiation of different antibiotics by guanidinylated polymyxins against (**a**) *P. aeruginosa* PAO1, (**b**) *A. baumannii* ATCC 17978 and (**c**) *E. coli* ATCC 25922. Fold potentiation was measured at 4 μg/mL of the adjuvant against *P. aeruginosa* PAO1 and 2 μg/mL of the adjuvant against *A. baumannii* ATCC 17978 and *E. coli* ATCC 25922. Fold potentiation ≥4 indicates synergy.

Following the assessment of the antibacterial activity, the ability of GCol and GPMB to synergize with a panel of 18 antibiotics was evaluated against wild-type Gram-negative bacteria. Antibiotics potentiated by at least four-fold were interpreted as synergistic. Against *P. aeruginosa* PAO1, GCol synergized with 16 antibiotics, while GPMB synergized with 15 antibiotics (Figure 2a). Against *A. baumannii* ATCC 17978, GCol synergized with four antibiotics, while GPMB synergized with eight antibiotics (Figure 2b). Against *E. coli* ATCC 25922, GCol synergized with 13 antibiotics, while GPMB synergized with 15 antibiotics (Figure 2c). The guanidinylated polymyxins, particularly GCol, did not synergize with most antibiotics against *A. baumannii* when compared at similar concentrations. However, at higher concentrations, both guanidinylated polymyxins consistently synergized with rifampicin, ceftazidime, clindamycin, aztreonam, piperacillin and pleuromutilin against all the wild-type strains tested (Table S1–S6). 

Notably, the MICs of several antibiotics in combination with the guanidinylated polymyxins reached comparable susceptibility breakpoints. Rifampicin (MICs of 0.008–0.5 μg/mL) reached its susceptibility breakpoint of 1 μg/mL in *Staphylococcus* spp. [25] against all the wild-type strains tested (Tables S1–S6). Erythromycin (MICs of 0.25 and 0.5 μg/mL) reached its susceptibility breakpoint of 0.5 μg/mL in *Enterococcus* spp. [25] against *E. coli* ATCC 25922 (Tables S5 and S6). Lastly, ceftazidime (MICs of 0.063–0.125 μg/mL) and aztreonam (MICs of 0.016–0.5 μg/mL) reached their susceptibility breakpoint of 8 μg/mL in *P. aeruginosa* and 4 μg/mL in Enterobacterales [25] against *P. aeruginosa* PAO1, *A. baumannii* ATCC 17978 and *E. coli* ATCC 25922 (Table S1–S6). These encouraging results provided the incentive to examine the potentiating effects of guanidinylated polymyxins against clinically relevant MDR clinical isolates of Gram-negative bacteria, strains defined to be resistant against one drug in at least three antibiotic classes [26]. 

### 2.4. Antibacterial Activity of GCol and GPMB against MDR Clinical Isolates of Gram-Negative Bacteria

The antibacterial activity of GCol and GPMB was assessed against MDR colistin-susceptible (Table 2) and colistin-resistant (Table 3) clinical isolates of Gram-negative bacteria prior to the evaluation of synergy. The MICs of GCol and GPMB were higher than colistin (≥32-fold) and PMB (≥4-fold) against colistin-susceptible *P. aeruginosa*, *A. baumannii* and *E. coli*, with the exception of GPMB, which retained the MIC of PMB against *A. baumannii* LAC-4 (Table 2). In general, GPMB possessed higher antibacterial activity than GCol against the colistin-susceptible clinical isolates. Moreover, both guanidinylated polymyxins showed increased antibacterial activity in comparison to PMBN.

The guanidinylated polymyxins also demonstrated higher or similar MICs compared to the respective parent compounds against colistin-resistant *P. aeruginosa*, *A. baumannii*, *E. coli*, *Enterobacter cloacae* and *Klebsiella pneumoniae*, with the exception of GPMB, which had a lower MIC than PMB against *P. aeruginosa* 114228 (Table 3). Both guanidinylated polymyxins showed comparable antibacterial activity to PMBN against the colistin-resistant clinical isolates, except *E. coli* 94474 and 94393, which was not the case against most of the colistin-susceptible strains.

### 2.5. Synergy of GCol and GPMB with Rifampicin against MDR Clinical Isolates of Gram-Negative Bacteria

**Figure 3:**
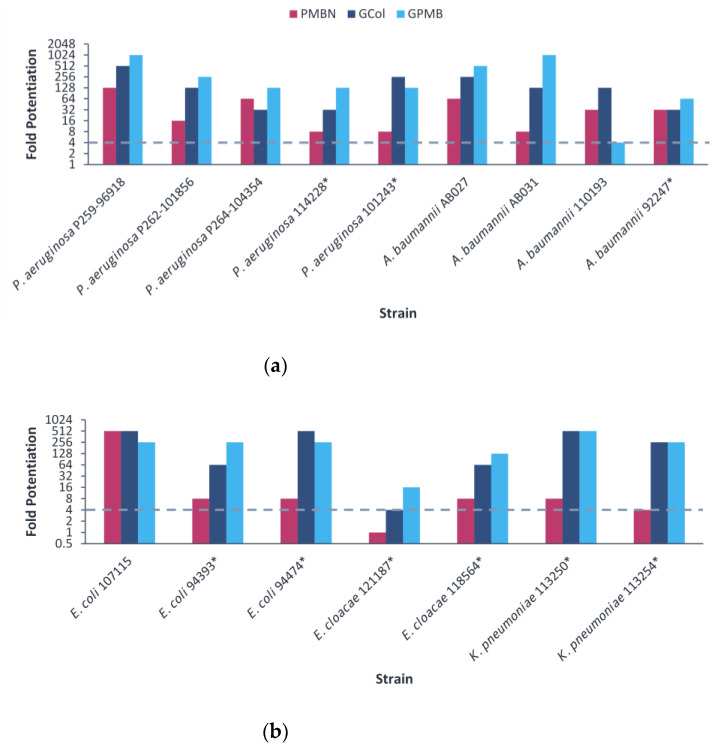
Potentiation of rifampicin by guanidinylated polymyxins and PMBN against clinical isolates of (**a**) *P. aeruginosa*, *A. baumannii*, (**b**) *E. coli*, *E. cloacae* and *K. pneumoniae*. The fold potentiation was measured at 8 μg/mL of the adjuvant, except for 4 μg/mL against *P. aeruginosa* P259-96918 and *E. coli* 94474, 2 μg/mL against *P. aeruginosa* P264-104354 and *E. coli* 94393, 1 μg/mL against *A. baumannii* 110193 and *E. coli* 107115 and 0.5 μg/mL against *A. baumannii* AB031. Fold potentiation ≥4 indicates synergy. * Colistin-resistant isolates.

The ability of GCol and GPMB, in comparison with PMBN, to potentiate rifampicin was then assessed against the MDR clinical isolates. The guanidinylated polymyxins retained synergy with rifampicin against all clinical isolates tested (Figure 3). In contrast, PMBN retained synergy with rifampicin against all the strains except *E. cloacae* 121187. The fold potentiation of rifampicin by the guanidinylated polymyxins remained higher than PMBN in most cases, particularly against *P. aeruginosa* P262-101856 and 101243, *A. baumannii* AB031, *E. coli* 94474 and *K. pneumoniae* 113250 and 113254 (Figure 3). Rifampicin in combination with the guanidinylated polymyxins (MICs of 0.001–1 μg/mL) or with PMBN (MICs of 0.031–1 μg/mL) reached the comparable susceptibility breakpoint of 1 μg/mL in *Staphylococcus* spp. [25] against most of the strains, except for *P. aeruginosa* P262-101856 (Table S7–S9).

### 2.6. Synergy of GCol and GPMB with Erythromycin against MDR Clinical Isolates of Gram-Negative Bacteria

**Figure 4:**
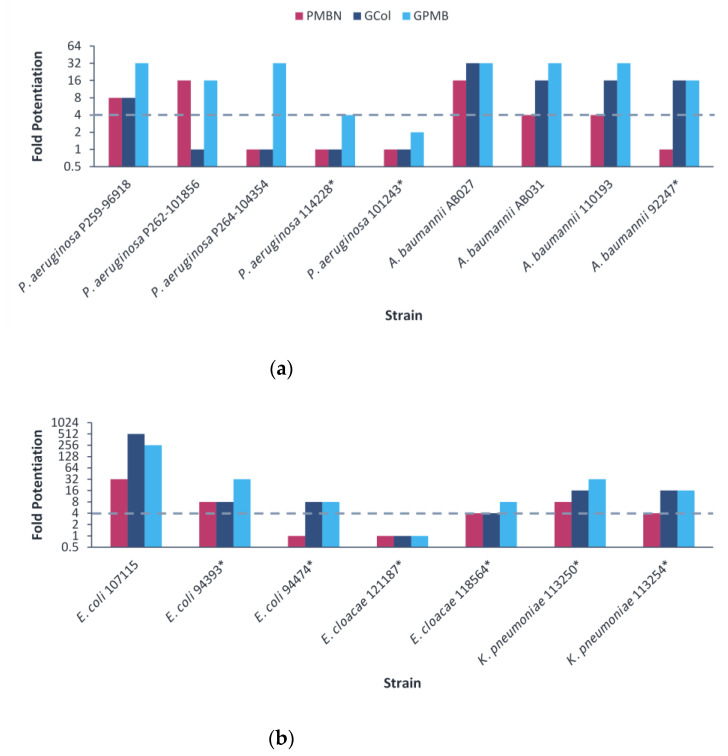
Potentiation of erythromycin by guanidinylated polymyxins and PMBN against clinical isolates of (**a**) *P. aeruginosa*, *A. baumannii*, (**b**) *E. coli*, *E. cloacae* and *K. pneumoniae*. The fold potentiation was measured at 8 μg/mL of the adjuvant, except for 4 μg/mL against *P. aeruginosa* P259-96918 and *E. coli* 94474, 2 μg/mL against *P. aeruginosa* P264-104354 and *E. coli* 94393, 1 μg/mL against *A. baumannii* 110193 and *E. coli* 107115 and 0.5 μg/mL against *A. baumannii* AB031. Fold potentiation of GCol against *P. aeruginosa* 262-101856 could not be determined at 8 μg/mL of the adjuvant. Fold potentiation ≥4 indicates synergy. * Colistin-resistant isolates.

The guanidinylated polymyxins retained synergy with erythromycin against several clinical isolates, with the exception of colistin-resistant *P. aeruginosa* and *E. cloacae* 121197 (Figure 4 and Table S10). In contrast, PMBN retained synergy with erythromycin against several strains, with the exception of colistin-resistant *P. aeruginosa, A. baumannii* and *E. coli* 94474 (Figure 4 and Table S10). The fold potentiation of erythromycin by the guanidinylated polymyxins were higher in comparison to PMBN against most of the *A. baumannii* strains. In addition, the fold potentiation of erythromycin by GPMB was higher than that of GCol against several *P. aeruginosa* strains. Erythromycin in combination with the guanidinylated polymyxins (MICs of 0.125–0.5 μg/mL) or with PMBN (MIC of 0.5 μg/mL) reached the comparable susceptibility breakpoint of 0.5 μg/mL in *Enterococcus* spp. [25] against several *A. baumannii* strains (Table S11). Meanwhile, erythromycin did not reach the susceptibility breakpoint in combination with either type of adjuvant against *P. aeruginosa* and *Enterobacteriaceae* (Tables S11 and S12).

### 2.7. Synergy of GCol and GPMB with Ceftazidime and Aztreonam against MDR Clinical Isolates of Gram-Negative Bacteria

**Figure 5:**
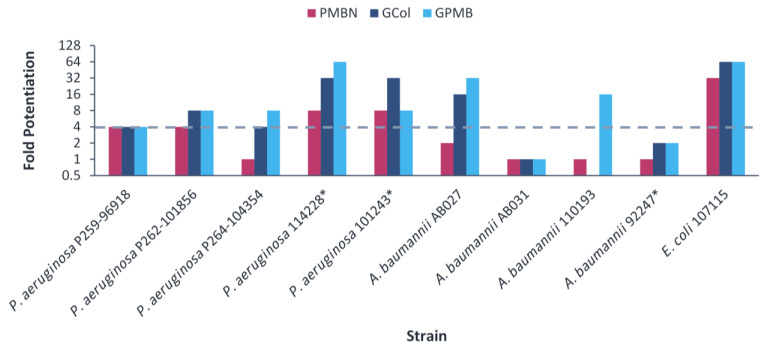
Potentiation of ceftazidime by guanidinylated polymyxins and PMBN against clinical isolates of *P. aeruginosa*, *A. baumannii* and *E. coli*. The fold potentiation was measured at 8 μg/mL of the adjuvant, except for 4 μg/mL against *P. aeruginosa* P259-96918, 2 μg/mL against *P. aeruginosa* P264-104354 and *E. coli* 107115, 1 μg/mL against *A. baumannii* 110193 and 0.5 μg/mL against *A. baumannii* AB031. Fold potentiation of GCol against *A. baumannii* AB031 could not be determined at 0.5 μg/mL of the adjuvant. Fold potentiation ≥4 indicates synergy. * Colistin-resistant isolates.

**Figure 6:**
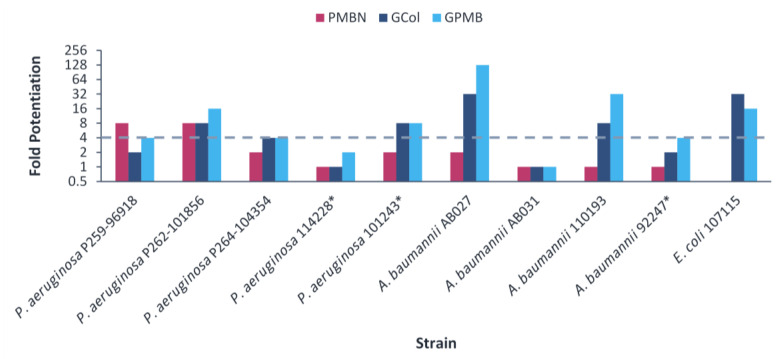
Potentiation of aztreonam by guanidinylated polymyxins and PMBN against clinical isolates of *P. aeruginosa*, *A. baumannii* and *E. coli*. The fold potentiation was measured at 8 μg/mL of the adjuvant, except for 4 μg/mL against *P. aeruginosa* P259-96918, 2 μg/mL against *P. aeruginosa* P264-104354 and *E. coli* 107115, 1 μg/mL against *A. baumannii* 110193 and 0.5 μg/mL against *A. baumannii* AB031. Fold potentiation of PMBN against *E. coli* 107115 could be determined at 2 μg/mL of the adjuvant. Fold potentiation ≥4 indicates synergy. * Colistin-resistant isolates.

The guanidinylated polymyxins retained synergy with both ceftazidime and aztreonam against all the clinical isolates of *P. aeruginosa* and had limited synergy against the clinical isolates of *A. baumannii* (Figure 5 and Figure 6). Similarly, PMBN lacked synergy with ceftazidime and aztreonam against clinical isolates of *A. baumannii*. The fold potentiation of ceftazidime and aztreonam by the guanidinylated polymyxins were comparable to PMBN against most of the strains. Ceftazidime and aztreonam in combination with the guanidinylated polymyxins (MICs of 0.25–8 μg/mL) reached the susceptibility breakpoint of 8 μg/mL in *P. aeruginosa* and *A. baumannii* [25] against most *P. aeruginosa* strains and a few *A. baumannii* strains (Tables S13–S16). In contrast, ceftazidime and aztreonam reached the susceptibility breakpoint in combination with PMBN (MICs of 0.5–8 μg/mL) only against *P. aeruginosa* strains (Tables S13–S16).

For the *E. coli* clinical isolates, synergy with ceftazidime and aztreonam was only assessed against *E. coli* 107115, since the two other strains were already susceptible to both β-lactam antibiotics. The guanidinylated polymyxins and PMBN retained synergy with both ceftazidime and aztreonam against *E. coli* 107115 (Figure 5 and Figure 6). However, ceftazidime or aztreonam in combination with GCol, GPMB or PMBN did not reach the susceptibility breakpoint of 4 μg/mL in Enterobacterales [25] (Table S17).

### 2.8. Triple Combination Studies against P. aeruginosa Harboring β-Lactamase Clinical Isolates

The ability of the guanidinylated polymyxins to synergize with ceftazidime and aztreonam was further examined against five *P. aeruginosa* clinical isolates harboring β-lactamases to determine whether the guanidinylated polymyxins would provide an additional benefit in the presence of these enzymes. The production of β-lactamases is supported by the increased susceptibility of these strains to ceftazidime in the presence of the β-lactamase inhibitor (BLI) avibactam (Tables S18–S21). The guanidinylated polymyxins maintained higher MICs than the respective parent compounds against *P. aeruginosa* clinical isolates harboring β-lactamases (Table 4). Since the guanidinylated polymyxins were active against *P. aeruginosa* PA 86052 and PA 108590, synergy was not assessed in this strain. 

The potentiation of ceftazidime and aztreonam with PMBN, GCol or GPMB was then investigated in the remainder of the *P. aeruginosa* clinical isolates harboring β-lactamases. Ceftazidime retained synergy with GCol and GPMB against *P. aeruginosa* PA 107092 and PA 109084 (Table S18). Aztreonam retained synergy with GPMB against *P. aeruginosa* PA 107092 and PA 109084 and with GCol against PA 107092 (Table S20). In contrast, both ceftazidime and aztreonam synergized with PMBN against *P. aeruginosa* PA 107092, PA 109084 and PA 86052. However, none of the three adjuvants were able to synergize with ceftazidime and aztreonam against PA 88949 (Tables S18 and S20).

The effect of introducing avibactam was also investigated to determine if the potency of ceftazidime and aztreonam could be further enhanced. The triple combination was assessed against *P. aeruginosa* PA 107092 and PA 109084, wherein synergy between ceftazidime and aztreonam with the guanidinylated polymyxins was observed.

**Figure 7:**
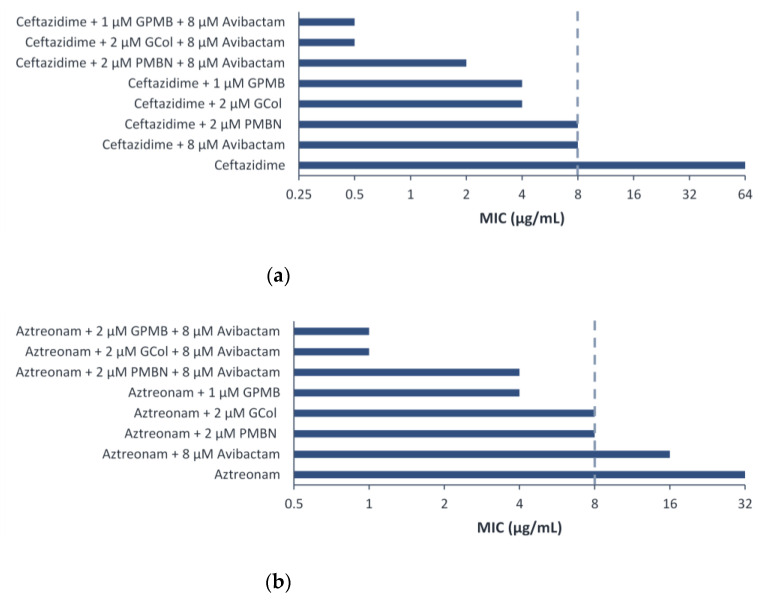
Triple combination of (**a**) ceftazidime and (**b**) aztreonam with avibactam and guanidinylated polymyxins or PMBN against *P. aeruginosa* PA 107092. The MIC of 8 μg/mL corresponds to the susceptibility breakpoint of ceftazidime and aztreonam against *P. aeruginosa*.

Ceftazidime retained synergy in the triple combination with avibactam and the guanidinylated polymyxins or PMBN against *P. aeruginosa* PA 107092 and PA 109084 (Table S19 and Figure S1a). In contrast, aztreonam retained synergy in the triple combination with avibactam and the guanidinylated polymyxins against *P. aeruginosa* PA 107092 and retained synergy with PMBN against *P. aeruginosa* PA 107092 and PA 109084 (Table S21 and Figure S1b). Ceftazidime in combination with the guanidinylated polymyxins or PMBN was potentiated eight-fold more (absolute MIC of 0.5 μg/mL) or four-fold more (absolute MIC of 1 μg/mL) than the dual combination against *P. aeruginosa* PA 107092, respectively (Figure 7a). On the other hand, aztreonam in combination with the guanidinylated polymyxins or PMBN was potentiated four- to eight-fold more (absolute MIC of 1 μg/mL) or two-fold more (absolute MIC of 4 μg/mL) than the dual combination against *P. aeruginosa* PA 107092, respectively (Figure 7b).

### 2.9. Time-Kill Kinetics Assay

To study the time-dependent killing effects of the drug combinations, the time-kill kinetics were assessed. The time-kill assay was performed following the 8 μM (2 μg/mL) concentration of avibactam used in the checkerboard assay. Furthermore, to follow the 4:1 clinical ratio of ceftazidime–avibactam, ceftazidime and aztreonam were used at a concentration of 8 μg/mL. This concentration also corresponds to the susceptibility breakpoint of both β-lactams [25]. GCol was selected as the lead compound based on its cytotoxicity profile and was used at a concentration of 2 μM, the corresponding concentration in the checkerboard assay wherein ceftazidime and aztreonam were potentiated. 

**Figure 8:**
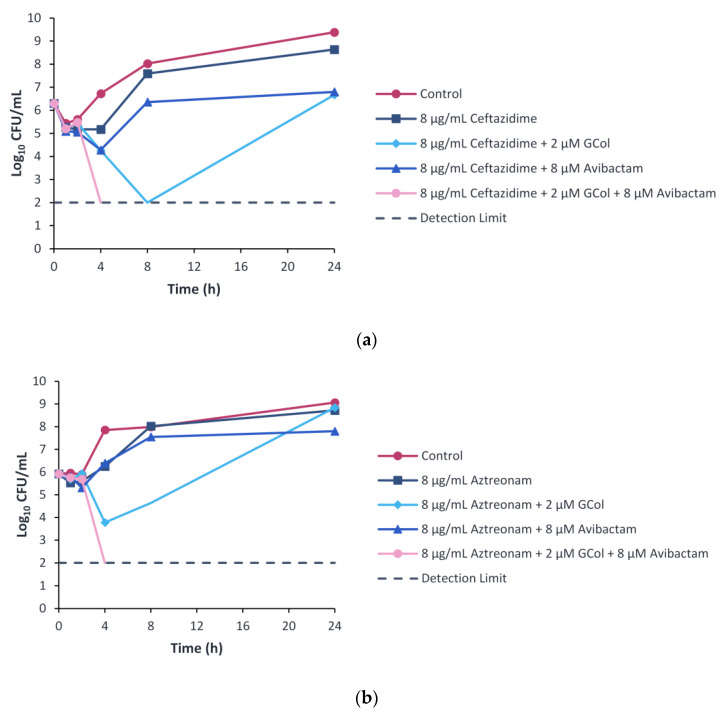
Time-kill curves of (**a**) ceftazidime and (**b**) aztreonam monotherapy, dual and triple combination with GCol and avibactam against *P. aeruginosa* PA 107092.

Ceftazidime monotherapy resulted in bacterial growth with a ~2 log_10_ increase over the 24-h period, similar to the control, while the dual therapy of ceftazidime with avibactam or GCol resulted in a bacteriostatic effect. The combination of the three components resulted in a rapid bactericidal effect with a ~4 log_10_ reduction within 4 h of treatment (Figure 8a). In addition, when the three agents were used at two-fold lower concentrations, bacterial growth was also observed (Figure S2). 

Aztreonam monotherapy and dual combination therapy with GCol or avibactam resulted in growth with a ~2 to 3 log_10_ increase over the 24-h time period, similar to the control. In contrast, the triple combination therapy of aztreonam, GCol and avibactam resulted in a rapid bactericidal effect with a ~4 log_10_ reduction within 4 h of treatment (Figure 8b).

### 2.10. OM Permeability Assay

**Figure 9:**
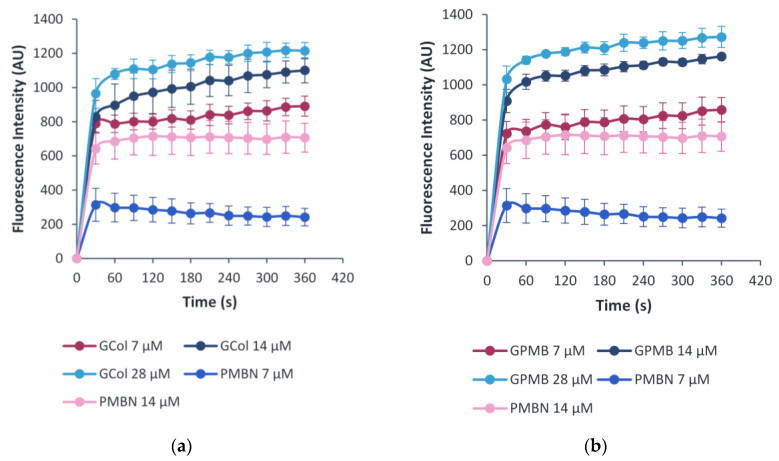
Measurement of OM permeabilization via NPN uptake induced by (**a**) GCol and (**b**) GPMB, with PMBN as a control against wild-type *E. coli* ATCC 25922 cells.

The ability of the guanidinylated polymyxins to permeabilize the OM in comparison to PMBN was evaluated through the uptake of 1-*N*-phenylnaphthylamine (NPN), a membrane-impermeable fluorophore. Permeabilization of the OM promotes the uptake of NPN in a time- and adjuvant concentration-dependent manner [27]. The change in fluorescence was measured as NPN fluoresces upon partitioning to the hydrophobic interior of the membrane [27]. The assay was done using wild-type *P. aeruginosa* PAO1, *A. baumannii* ATCC 17978 and *E. coli* ATCC 25922 cells (Figure 9, Figures S3 and S4). Increasing concentrations of the guanidinylated polymyxins and PMBN correlated with an increase in the observed fluorescence, indicating NPN uptake in an OM permeabilizer concentration-dependent manner.

### 2.11. Cytotoxicity Assay

**Figure 10:**
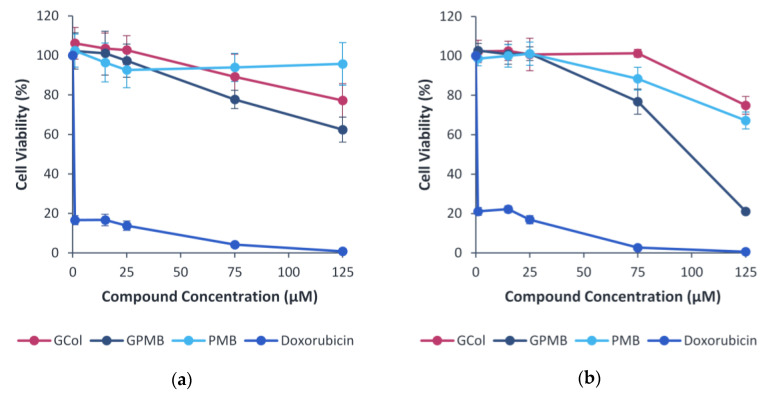
Cytotoxicity of the guanidinylated polymyxins in (**a**) HEK293 and (**b**) Hep G2 cells. PMB and doxorubicin were used for comparison.

As an initial screening of the toxicity, the effect of the guanidinylated polymyxins, in comparison with PMB and doxorubicin, on the viability of human embryonic kidney cells (HEK293) (Figure 10a) and liver carcinoma cells (Hep G2) (Figure 10b) cells was evaluated. In the HEK293 cells, GCol was less cytotoxic than GPMB, but GCol was more cytotoxic than PMB at higher concentrations. HEK293 cells maintained >90% viability up to 125 μM (173 μg/mL) of PMB and only up to 25 μM of either GCol (48 μg/mL) or GPMB (50 μg/mL). At 125 μM, HEK293 viability decreased to 77.2% and 62.4% for GCol (242 μg/mL) and GPMB (248 μg/mL), respectively.

In the Hep G2 cells, GCol was less cytotoxic than GPMB and PMB. Hep G2 cells maintained 100% viability up to 75 μM (145 μg/mL) of GCol and only up to 25 μM of GPMB (50 μg/mL) and PMB (35 μg/mL). At 125 μM, Hep G2 viability decreased to 74.9% and 67.2% for GCol (242 μg/mL) and PMB (173 μg/mL), respectively. GPMB was significantly more cytotoxic, reducing Hep G2 viability to 21.1% at 125 μM (248 μg/mL).

## 3. Discussion

In comparison to their parent polymyxins, GCol and GPMB exhibited higher MICs against the wild-type and colistin-susceptible isolates and demonstrated similar or higher MICs against the colistin-resistant clinical isolates, with a few exceptions (Table 1, Table 2 and Table 3). These results suggest that the conversion of the Dab amines to guanidium groups results in reduced antibacterial activity. This is consistent with previously synthesized polymyxins analogs with derivatized Dab side chains in an SAR study, which highlights the specificity of the Dab residues [15]. Among the five Dab residues, alterations of the Dab at position 5 resulted in a substantial loss of antibacterial activity [28]. However, GCol and GPMB demonstrated lower MICs when compared to PMBN in some cases, suggesting that the antibacterial activity is retained to some extent, in part due to the presence of the lipid tail. 

Utilized as adjuvants, the guanidinylated polymyxins synergized with different classes of antibiotics against wild-type Gram-negative bacteria. The guanidinylated polymyxins synergized with OM-impermeable antibiotics, such as rifampicin, novobiocin and erythromycin, against all wild-type organisms (Figure 2). Moreover, GPMB synergized with vancomycin, clindamycin and linezolid against wild-type bacteria (Figure 2). The potentiation of such antibiotics is a key characteristic of OM permeabilizers and has been extensively reported with other adjuvants. In contrast, the predominant lack of synergy with OM-permeabilizing antibiotics such as tobramycin and colistin was predicted. Aminoglycosides and polymyxins, which share a similar mechanism of self-promoted uptake, are both capable of disrupting the OM [15,29,30] and will not benefit from enhanced OM permeability. It is also possible that a combination of OM permeabilizers could result in both agents competing for LPS binding. In addition, the guanidinylated polymyxins synergized with antibiotics that enter the cell through porins, such as ceftazidime, aztreonam, piperacillin, ciprofloxacin, levofloxacin, moxifloxacin, minocycline, doxycycline and chloramphenicol (Figure 2). This suggests that the use of an OM permeabilizer effectively increases the periplasmic and/or intracellular concentration of these antibiotics. However, only the potentiation of the β-lactams was consistently observed against all the wild-type strains tested, while the potentiation of the fluoroquinolones, tetracyclines and chloramphenicol were limited to *P. aeruginosa* PAO1 and displayed variable success against *A. baumannii* ATCC 17978 and *E. coli* ATCC 25922 (Tables S1–S6). The limited synergy is likely due to the efflux-susceptibility of these antibiotics. These results, along with the overall reduction in the number of antibiotics potentiated in wild-type *A. baumannii* and *E. coli*, in comparison to *P. aeruginosa*, could also be attributed to variations of intrinsic resistance mechanisms between the strains. The intrinsic low OM permeability of *P. aeruginosa* is the main factor affecting influx of antibiotics [31,32]. Therefore, permeabilization of the OM of *P. aeruginosa* by the guanidinylated polymyxins has a considerable effect on the potentiation of these antibiotics. In contrast, potentiation of antibiotics using porin-mediated uptake in wild-type *A. baumannii* is challenging and is likely the result of the various intrinsic efflux systems in *A. baumannii* [33]. Nonetheless, the guanidinylated polymyxins potentiated both OM impermeable and porin-mediated antibiotics, notably rifampicin, erythromycin, ceftazidime and aztreonam, against wild-type Gram-negative bacteria. The potentiating effects of the guanidinylated polymyxins in combination with these antibiotics were then further investigated in clinically relevant MDR isolates. 

Rifampicin is used in the treatment of *Mycobacterium* infections and is also active against some Gram-positive bacteria, such as methicillin-resistant *Staphylococcus aureus* [34]. Rifampicin is inactive against Gram-negative bacteria due to its inability to transit the OM [35]. The high hydrophobicity and large molecular weight of rifampicin limit both passive diffusion along the lipid bilayer and entry through porins that selectively allow the uptake of small, hydrophilic molecules [32]. Rifampicin in combination with GCol and GPMB afforded low MICs, ranging from 0.004–0.063 μg/mL and 0.001–0.063 μg/mL, respectively, against colistin-susceptible and resistant *P. aeruginosa* and *A. baumannii* and colistin-susceptible *E. coli* (Tables S7–S9). In contrast, rifampicin in combination with PMBN had MICs ranging from 0.031–0.063 μg/mL (Tables S7–S9). Thus, rifampicin/guanidinylated polymyxins combinations were able to lower the MICs to a level where they restored susceptibility in previously resistant isolates. 

Erythromycin is a macrolide antibiotic, active against Gram-positive bacteria, and it has limited activity against Gram-negative bacteria [36]. Analogous to rifampicin, it is incapable of crossing the OM due to its hydrophobicity and high molecular weight [36,37]. Erythromycin in combination with the guanidinylated polymyxins attained its comparable susceptibility breakpoint of 0.5 μg/mL against *Enterococcus* spp., with MICs of 0.125 and 0.25 μg/mL, against wild-type *E. coli* (Tables S5 and S6). Erythromycin retained synergy with the guanidinylated polymyxins against most of the clinical isolates tested, particularly losing its synergy against colistin-resistant *P. aeruginosa* (Figure 4). Erythromycin/guanidinylated polymyxin combinations were able to lower the MICs to a level where they restored susceptibility in colistin-resistant *A. baumannii* (Tables S10–S12). The MICs of erythromycin in combination with GCol and GPMB were only reduced to 0.125–0.25 μg/mL against two colistin-susceptible *A. baumannii* strains (Table S11). In contrast, erythromycin in combination with PMBN reached slightly higher absolute MICs (0.5 μg/mL) against these strains (Table S11). 

Ceftazidime and aztreonam are β-lactam antibiotics. Ceftazidime is a third-generation cephalosporin with broad spectrum activity, particularly against *P. aeruginosa* [38,39]. Aztreonam is a monobactam, a subgroup among β-lactam antibiotics, potent against aerobic Gram-negative bacteria, including *P. aeruginosa* [40]. Synergy determination was omitted against the colistin-resistant *E. coli*, *E. cloacae* and *K. pneumoniae* clinical isolates due to the susceptibility of these strains to ceftazidime and aztreonam. Nonetheless, ceftazidime and aztreonam retained synergy with the guanidinylated polymyxins against most of the remaining clinical isolates (Figure 5 and Figure 6) and reached the susceptibility breakpoint against several *P. aeruginosa* and *A. baumannii* strains (Tables S13–S16). In addition, the potentiation of these antibiotics was significantly higher in comparison to the wild-type strains, particularly *P. aeruginosa* PAO1 and *E. coli* ATCC 25922. This could be attributed to the susceptibility of the wild-type organisms to these antibiotics and that increased intracellular uptake would have a more substantial effect on the MDR clinical isolates, wherein permeability would be limited. 

However, aztreonam and ceftazidime are vulnerable to hydrolysis by β-lactamases. The emergence and increasing dissemination of these β-lactamase enzymes worldwide [41], specifically the metallo-β-lactamases (MBLs), are a particular concern due to the lack of effective treatment options [41,42]. Although aztreonam is stable against MBLs in contrast to ceftazidime [40], it is still inactivated by serine enzymes such as various extended-β-lactamases (ESBLs), *K. pneumoniae* carbapenemases (KPCs) and AmpC cephalosporinases, which may be produced along with MBLs [40,43]. β-Lactam antibiotics are frequently used in combination with BLIs. 

Avibactam is a BLI that reversibly inhibits [44] ESBLs, KPCs and cephalosporinases [45] and has been used clinically in combination with ceftazidime, restoring its activity against these enzymes [43]. Avibactam in combination with aztreonam is currently in clinical development [46] and has demonstrated its efficacy as a potential therapeutic option, particularly against MBL-producing Enterobacterales [47]. Therefore, it is relevant to examine whether the guanidinylated polymyxins could still enhance the potency of aztreonam and ceftazidime in the presence of β-lactamases, and if it would provide an additional benefit in a triple combination therapy with avibactam.

Ceftazidime and aztreonam retained synergy with the guanidinylated polymyxins against several *P. aeruginosa* clinical isolates harboring β-lactamases, reducing the MIC to the susceptibility breakpoint in most cases (Tables S18 and S20). In contrast, ceftazidime and aztreonam retained synergy with PMBN against the majority of the isolates tested, reducing the MIC to the susceptibility breakpoint consistently (Tables S18 and S20). The limited synergy between the β-lactams and the guanidinylated polymyxins was ascribed to the low MICs of these adjuvants against some of the strains. Against the strains wherein synergy between the guanidinylated polymyxins and ceftazidime or aztreonam was observed, the inclusion of avibactam as a third component further enhanced the potency of the β-lactams (Tables S19 and S21 and Figure S1). The results from the time-kill curves revealed that the dual combinations of guanidinylated polymyxins with ceftazidime or aztreonam did not induce bactericidal effects (Figure 7). In contrast, the synergistic relationship of the triple combinations of either ceftazidime or aztreonam with GCol and avibactam in the checkerboard assays translated to a rapid bactericidal effect within 4 hours of treatment in the time-kill experiments (Figure 8). The effect also appeared to be dose-dependent, as two-fold lower concentrations of each agent were not sufficient to elicit bactericidal effects (Figure S2). The addition of avibactam proved to be a necessity, as the saturation of β-lactamase enzymes in the periplasm possibly reduces the activity or renders the β-lactams inactive despite increased accumulation of these antibiotics from diminished OM permeability. It has also been theorized that permeabilization of the OM could consequently cause leakage of the β-lactamases from the periplasm [12].

In addition to the possible clinically relevant applications of the guanidinylated polymyxins, it is also valuable to elucidate any SAR that could be derived from their potentiation effects in comparison to PMBN, specifically distinguishing between the colistin-susceptible and -resistant clinical isolates. 

Rifampicin was significantly potentiated by the guanidinylated polymyxins in comparison to PMBN against colistin-resistant clinical isolates of *P. aeruginosa*, *E. coli* and *K. pneumoniae* (Figure 3). In contrast, rifampicin was potentiated comparably by both the guanidinylated polymyxins and PMBN against most strains of colistin-susceptible *P. aeruginosa* and *E. coli* (Figure 3). Since the potentiation of rifampicin is predominantly tied to its inability to cross the OM, these results most likely occur due to differences in the OM. The colistin-resistant *E. coli* strains used in this study contain the *mcr*-1 gene. This plasmid-mediated gene encodes phosphoethanolamine transferase, which results in the alteration of the LPS via incorporation of a positively charged phosphoethanolamine to lipid A, thereby decreasing the binding affinity of polymyxins through electrostatic repulsion [48]. While the underlying colistin-resistance mechanisms in the *P. aeruginosa* and *K. pneumoniae* strains are unknown, various routes leading to LPS modifications by addition of either 4-amino-4-deoxy-L-arabinose or phosephoethanolamine are evident [49]. These results coincide with the reduced LPS binding of polymyxins against *mcr*-1 positive *E. coli* and the decreased uptake of membrane-impermeable NPN in *P. aeruginosa* and *A. baumannii* strains with chromosomally encoded aminoarabinose and phosphoethanolamine [27]. LPS modifications likely affect the degree of potentiation by the guanidinylated polymyxins and PMBN. The heightened potentiation by the guanidinylated polymyxins presumably arise from factors such as increased membrane expansion facilitated by the hydrophobic lipid tail, increased number of positive charges and the higher basicity imparted by the guanidine functions.

Conversely, the observed trend in rifampicin potentiation was not evident in erythromycin, despite also being restricted by the permeability barrier. Erythromycin was only significantly potentiated by the guanidinylated polymyxins in comparison to PMBN against colistin-resistant *A. baumannii* and was potentiated comparably by both the guanidinylated polymyxins and PMBN against colistin-susceptible *A. baumannii* (Figure 4). Moreover, erythromycin demonstrated reduced potentiation by both the guanidinylated polymyxins and PMBN against colistin-resistant *P. aeruginosa* and *E. coli*, in comparison to the colistin-susceptible counterparts (Figure 4). This is in part caused by resistance mechanisms targeting erythromycin, as opposed to strictly changes in the OM. Mutations on the 23S rRNA and methylase genes confer resistance to macrolides in Gram-negative bacteria [50,51]. In addition, the intrinsic and acquired MexAB-OprM efflux system in *P. aeruginosa* has also been demonstrated to extrude macrolide antibiotics outside of the cell [52]. As a result, an increased intracellular concentration from decreased OM permeability by the guanidinylated polymyxins and PMBN may not have a profound effect in circumventing active site modifications and overexpression of efflux pumps. Nevertheless, the guanidinylated polymyxins and PMBN appeared to be equally affected by LPS modifications in *P. aeruginosa* and *E. coli*. However, the potentiating effects related to the structural differences between the guanidinylated polymyxins and PMBN may not be apparent. 

Correspondingly, no consistent trends related to colistin-resistance were apparent in the potentiation of ceftazidime and aztreonam by the guanidinylated polymyxins (Figure 5 and Figure 6). This is because the potentiation of the β-lactam antibiotics is not entirely dependent on changes in the OM, as they also have an alternative mode of uptake, but it is rather considerably influenced by the presence of β-lactamases. 

Additional evidence pertaining to the ability of the guanidinylated polymyxins to preserve the ability to permeabilize the OM despite loss of standalone activity was supported by the OM permeabilization assay. The studies conducted in wild-type *P. aeruginosa* PAO1, *A*. *baumannii* ATCC 17978 (Figures S9 and S10) and *E. coli* ATCC 25922 (Figure 9) demonstrated that the guanidinylated polymyxins allowed uptake of NPN similar to PMBN, an established OM permeabilizer. 

Lastly, the inherent nephrotoxicity and neurotoxicity of the polymyxins is one of the main challenges that limit clinical application. The toxic effects have been attributed to the polycationic nature of these compounds [53,54]. A reduction in the net positive charge as well as alterations or removal of the fatty acyl chain have been correlated to reduced polymyxin-induced nephrotoxicity, evidenced by both PMBN and SPR741, which are markedly less nephrotoxic in comparison to PMB [55]. However, changing the nature of all the groups imparting cationicity and its influence on the toxicity has not yet been examined. As a preliminary screening of the toxicity, the cytotoxicity of the guanidinylated polymyxins in comparison to PMB was assessed in HEK293 and Hep G2 cells. In HEK293, PMB was less cytotoxic than the guanidinylated polymyxins at higher concentrations (Figure 10a). In Hep G2, GCol was less cytotoxic than GPMB and PMB (Figure 10b). GPMB was more cytotoxic than its precursor in both cell lines, suggesting that perhaps conversion of the amines to a guanidine function may not eliminate the toxicity. However, these initial results are not indicative of polymyxin-induced nephrotoxicity. The cytotoxicity assay conducted in these cells is weakly correlated with experiments in human kidneys, which are more accurate predictors of nephrotoxicity [28]. Follow-up toxicity studies should utilize biomarkers of nephrotoxicity to conclude the effects of guanidinylation. Nonetheless, cell viability was maintained at significantly higher concentrations than the requirement for synergy.

To summarize, colistin and PMB were repurposed as effective OM permeabilizers by direct conversion to their guanidinylated analogs through a two-step synthetic route. The substitution of the primary amines of the Dab residues to guanidinium groups resulted in reduced antibacterial activity but conserved the inherent ability of polymyxins to permeabilize the OM. Utilized as adjuvants, GCol and GPMB synergized with various antibiotic classes, particularly improving the activity of rifampicin, erythromycin, ceftazidime and aztreonam. In the case of rifampicin and erythromycin, the MICs in combination with the guanidinylated polymyxins were reduced below the susceptibility breakpoint against several multidrug-resistant clinical isolates, indicating that these adjuvant/antibiotic combinations restored susceptibility in previously resistant isolates. On the other hand, the potency of ceftazidime and aztreonam were greatly enhanced with the inclusion of the guanidinylated polymyxins in a triple combination therapy with avibactam against β-lactamase harboring *P. aeruginosa*. Moreover, GCol and GPMB demonstrated enhanced OM permeabilizing capabilities in comparison to PMBN, as evidenced by the higher potentiation effects in most cases, and resulted an increased uptake of NPN. The guanidinium groups presumably play a role, although the increased number of positive charges and presence of the lipid tail could also contribute to increased OM permeability However, preliminary studies suggest that guanidinylation may not lessen the toxicity associated with the use of polymyxins. Thus, further studies should assess the toxicity profile of these compounds in more depth.

## 4. Materials and Methods

### 4.1. Preparation of Guanidinylated Polymyxins GPMB and GCol 

All reagents and chemicals were purchased from Sigma-Aldrich (St. Louis, MO, USA), except for PMB sulfate purchased from AK Scientific, Inc. (Union City, CA, USA) and *N,N′*-bis(tert-butoxycarboyl)-*N″*-trifylguanidine purchased form Biosynth Carbosynth (San Diego, CA, USA). All reagents and chemicals were used without further purification. Colistin and PMB are natural products that exist in two lipid forms referred to as colistin A and B and PMB_1_ and PMB_2_, respectively. 

The guanidinylated polymyxins were synthesized following established methods [24] with minor adjustments. Colistin (0.039 mmol) or PMB (0.038 mmol) was dissolved in 0.5 mL water. *N,N′*-Bis(tert-butoxycarboyl)-*N″*-trifylguanidine (0.59 mmol, 15 molar eq.) and 1,4-dioxane (2.5 mL) were then added in alternating portions to ensure that the solution remained relatively clear. After 5 min, triethylamine (0.082 mL, 0.59 mmol, 15 molar eq.) was added at room temperature. After 3–4 days, 1,4-dioxane was removed under reduced pressure. The remaining residue was extracted thrice with dichloromethane (DCM). The organic layers were washed with brine and dried using anhydrous sodium sulfate. The tert-butyloxycarbonyl (Boc)-protected guanidinylated polymyxins were then purified via normal phase flash chromatography using silica gel (40–63 μm) from Silicycle (Quebec, QC, Canada) and eluted with 60:1 to 30:1 DCM/methanol (*v*/*v*). The guanidinylation yielded 59% of Boc-protected GCol and 68% of Boc-protected guanidinylated GPMB. 

For the removal of the Boc-protecting groups, 2 mL of 1:1 trifluoroacetic acid (TFA)/DCM (*v*/*v*) was added to the protected guanidinylated polymyxins. After 1 hour, TFA and DCM were removed under reduced pressure. Then, 2% methanol in ether (*v*/*v*) was added to the remaining residue, stirred for 1 min and the solvent decanted. The crude products were then purified via reverse-phase flash chromatography using C_18_ silica gel (40–63 μm) from Silicycle (Quebec, QC, Canada) and eluted with 100% water to 20% methanol in water spiked with 0.1% TFA (*v*/*v*). The deprotection yielded 86% of GCol and 90% GPMB as TFA salts. Both polymyxins were obtained as a ratio of the two lipopeptides that differ by one carbon atom in the lipid portion. 

The guanidinylated polymyxins were characterized using 1D and 2D nuclear magnetic resonance (NMR) spectroscopy experiments, such as ^1^H, ^13^C, COSY, HSQC, HMBC and DEPT-135 (Figures S5–S16) on Bruker AMX-300 and AMX-500 spectrometers (Germany). The molecular weights of the guanidinylated polymyxins were confirmed using matrix-assisted laser desorption/ionization mass spectrometry (MALDI-MS) in positive ion mode with 2,5-dihydroxybenzoic acid as the matrix on Bruker Ultraflextreme (Germany). The mass spectra indicated the presence of both guanidinylated colistin A and B and guanidinylated PMB_1_ and PMB_2_ (Figures S17 and S18). The purity of the guanidinylated polymyxins was assessed using high-performance liquid chromatography (HPLC) on a Thermo Scientific Vanquish Ultra-HPLC (Waltham, MA, USA) with a Phenomenex (Torrance, CA, USA) Kinetex 2.6 μm XB-C18 100 Å, 100 × 4.6 mm reverse-phase column based on previously developed methods with adjustments [56]. The chromatograms indicated ≥95% purity and the relative percentages of the major components of the guanidinylated polymyxins. GCol consisted of 58% GCol A and 42% GCol B, while GPMB was comprised of 88% GPMB_1_ and 12% GPMB_2_ (Figures S19 and S20).

#### 4.1.1. Chemical Characterization of GCol

^1^H NMR (500 MHz, D_2_O) δ 4.37–4.34 (m, 1H guanidinylated Dab_5α_), 4.31–4.26 (m, 2H, guanidinylated Dab_1,8α_), 4.21–4.06 (m, 8H, Thr_2α_, guanidinylated Dab_3,9α_ + Dab_4α_ + Thr_2β_ + Thr_10β_, Leu_6α_, Leu_7α_), 4.02–4.01 (m, 1H, Thr_10α_), 3.27–3.07 (m, 12H, guanidinylated Dab_1,3,5,8,9γ_ + Dab_4γ_), 2.19–2.16 (m, 2H, a, aliphatic), 2.04–1.75 (m, 12H, guanidinylated Dab_3,5β1_ + guanidinylated Dab_3,5β2_ + guanidinylated Dab_1,8,9β_ + Dab_4β_), 1.55–1.39 (m, 9H, Leu_6,7β_, Leu_6,7γ_, b, e, aliphatic), 1.15–1.10 (4H, c + d, aliphatic), 1.07–1.04 (m, 6H, Thr_2γ_ + Thr_10γ_), 1.03–0.92 (m, 2H, g, aliphatic), 0.79–0.77 (m, 6H, Leu_6δ_), 0.75–0.71 (m, 6H, h + f, aliphatic) and 0.69–0.59 (m, 6H, Leu_7δ_).

^13^C NMR (126 MHz, D_2_O) δ 177.92, 175.08, 174.99, 174.20, 173.93, 173.47, 172.99, 172.86, 172.76, 171.83, 163.24, 162.96, 162.67, 162.39, 156.92, 156.83, 119.78, 117.46, 115.15, 112.83, 66.94 (Thr_2β_), 66.26 (Thr_10β_), 59.56 (Thr_10α_), 59.14 (Thr_2α_), 53.33, 52.13, 51.82, 51.53, 50.81 (guanidinylated Dab_5α_), 39.54, 38.91 (aliphatic, b), 37.90, 37.82, 36.16, 35.49 (a, aliphatic), 33.59, 30.90, 29.75, 29.51, 28.90, 27.16, 26.13, 25.62, 25.55, 24.43, 22.48, 21.91, 21.79, 21.10, 20.02, 19.21, 18.83, 18.58 and 10.72.

MS (+TOF) *m*/*z*: calculated for [M+H]^+^ GCol A and B: 1379.88 and 1365.87, found: 1379.87 and 1365.85; [M+Na]^+^ GCol A and B: 1401.86 and 1387.85, found: 1401.85 and 1387.83.

#### 4.1.2. Chemical Characterization of GPMB

^1^H NMR (500 MHz, D_2_O) δ 7.25–7.16 (m, 3H, Phe_6_, aromatic), 7.12–7.10 (d, 2H, Phe_6_, aromatic), 4.39–4.36 (m, 1H, Phe_6α_), 4.33–4.26 (m, 3H, guanidinylated Dab_1,5,8α_), 4.18–4.17 (m, 1H, Thr_2α_), 4.14–4.08 (m, 5H, guanidinylated Dab_3,9α_ + Dab_4α_ + Thr_2β_ + Thr_10β_), 4.03–4.00 (2H, m, Thr_10α_ + Leu_7α_), 3.26–3.03 (10H, m, guanidinylated Dab_1,3,8,9γ_ + Dab_4γ_), 3.02–2.87 (m, 4H, Phe_β_ + guanidinylated Dab_5γ_), 2.19–2.16 (m, 2H, a, aliphatic), 2.04–1.63 (m, 11H guanidinylated Dab_3,5β1_ + guanidinylated Dab_3,5β2_ + guanidinylated Dab_1,8,9β_ + Dab_4β_), 1.46–1.40 (m, 2H, b, aliphatic), 1.37–1.30 (m, 1H, Leu_7β1_), 1.27–1.22 (m, 1H, Leu_7β2_), 1.15–1.09 (m, 5H, c + d + e, aliphatic, Leu_7γ_), 1.06–1.02 (m, 6H, Thr_2γ_ + Thr_10γ_), 1.00–0.92 (m, 2H, g, aliphatic), 0.69–0.65 (m, 6H, h + f, aliphatic), 0.60 (m, 3H, Leu_7δ_) and 0.53–0.51 (m, 3H, Leu_7δ_).

^13^C NMR (126 MHz, D_2_O) δ 177.93, 175.14, 174.11, 173.94, 173.53, 173.47, 172.99, 172.81, 172.33, 171.80, 171.62, 163.36, 163.08, 162.80, 162.52 (TFA, carbonyl), 156.91, 156.88, 156.83, 156.72, 135.51 (Phe_6_, aromatic with no proton), 129.04, 127.49 (Phe_6_, aromatic), 119.87, 117.55, 115.23, 112.91 (TFA, trifluoromethyl), 66.94 (Thr_2β_), 66.18 (Thr_10β_), 59.69 (Thr_10α_), 59.13 (Thr_2α_), 56.11 (Phe_6α_), 52.77, 52.03, 51.90 (Leu_7α_), 51.67, 51.55, 51.35, 50.63, 46.75, 39.12 (Leu_7β_), 37.97, 37.91, 37.81, 37.53, 36.80, 36.08, 35.49, 35.44 (a, aliphatic), 33.60, 30.92, 30.05, 29.91, 29.67, 29.38, 28.90, 25.98, 25.80, 25.63 (b, aliphatic), 23.51, 22.43 (Leu_7δ_), 21.98, 21.92, 20.32 (Leu_7δ_), 19.21, 18.83, 18.58 and 10.73 (h, aliphatic).

MS (+TOF) *m*/*z*: calculated for [M+H]^+^ GPMB_1_ and B_2_: 1413.87 and 1399.85, found: 1413.87 and 1399.85; [M+Na]^+^ GCol A and B: 1435.85 and 1421.83, found: 1435.89 and 1421.87.

### 4.2. Bacterial Isolates and Growth Conditions

All antibiotics were purchased from commercial sources. Bacterial isolates were obtained from the American Type Culture Collection (ATCC) and from the Canadian National Intensive Care Unit (CAN-ICU) [57] and Canadian Ward (CANWARD) surveillance studies [58]. CAN-ICU and CANWARD clinical isolates were collected from patients with presumed infectious diseases admitted in participating Canadian medical centers either to the intensive care unit or medical wards. Prior to microbiological testing, bacterial cultures were grown overnight in lysogeny broth (LB) at 37 °C, shaking at 250 rpm.

### 4.3. Antimicrobial Susceptibility Assay

The antibacterial activities of GPMB and GCol were evaluated using the broth microdilution method, in accordance with standard protocols, as previously described [10]. For the preparation of the bacterial solution, overnight grown culture was diluted in 0.85% saline solution, adjusted to 0.5 McFarland turbidity and further diluted in Mueller–Hinton broth for inoculation to a final concentration of 5 × 10^5^ colony forming units/mL (CFU/mL). The agents were serially diluted two-fold in a 96-well plate, and equal volumes of bacterial solution were subsequently added to the designated wells. The well consisting of bacterial solution served as the positive control, while the well containing only media served as the negative control. The plate was then incubated at 37 °C for 18 h. After incubation, an Emax Plus microplate reader (Molecular Devices, Union City, CA, USA) was used to measure the optical density (OD) at a wavelength of 590 nm to confirm the turbidity. The antibacterial activity corresponded to the MIC, which is defined as the lowest concentration of the agent necessary to inhibit visible bacterial growth.

### 4.4. Checkerboard Assay

The adjuvant properties of the guanidinylated polymyxins were evaluated using the checkerboard assay as previously described [10]. The bacterial solution was prepared as described in the antimicrobial susceptibility assay. The antibiotic of interest and the adjuvant were serially diluted two-fold along the *x-* and *y*-axis on a 96-well plate, respectively, resulting in varying concentrations of both agents in each well. Equal volumes of bacterial solution were added to the designated wells. The well consisting of bacterial solution served as the positive control, while the wells containing only media served as the negative control. The plate was then incubated at 37 °C for 18 h. After incubation, an Emax Plus microplate reader (Molecular Devices, Union City, CA, USA) was used to measure the OD at a wavelength of 590 nm to confirm the turbidity. The fractional inhibitory concentration (FIC) index was then determined to establish the relationship between the antibiotic and adjuvant. The FIC index corresponds to the sum of the FICs of the antibiotic and adjuvant. The FIC of each agent is calculated by dividing its MIC when it is used in combination by its MIC when used alone. FIC indices ≤0.5, 0.5 < *x* ≤ 4 and >4 indicate synergy, additivity and antagonism, respectively.

### 4.5. Time-Kill Assay

The bacteriostatic or bactericidal activities of ceftazidime and aztreonam in dual and triple combinations with GCol and avibactam were evaluated using the time-kill assay as previously described [9]. For the preparation of the bacterial solution, an overnight grown culture was diluted in 0.85% saline solution and adjusted to 0.5 McFarland turbidity. A total of 60 μL of the resulting inoculum was further diluted in 3 mL of lysogeny broth (LB) containing different combinations of either ceftazidime or aztreonam, with GCol and avibactam. The culture tubes were incubated at 37 °C, shaking at 250 rpm. At the appointed time intervals, a 100-μL aliquot was taken from each tube, serially diluted in phosphate-buffered saline (PBS) and plated on LB agar plates. The plates were incubated at 37 °C, and bacterial colonies were counted after 18 h. The bactericidal or bacteriostatic activity is determined based on the decrease in CFU over the time period. Bactericidal activity corresponds to ≥3 log_10_ reduction in CFU/mL, while bacteriostatic activity corresponds to <3 log_10_ reduction in CFU/mL.

### 4.6. OM Permeabilization Assay

The OM permeabilizing properties of the guanidinylated polymyxins were evaluated using the NPN uptake assay based on previously established protocols [27] with minor modifications. For the preparation of the bacterial cells, overnight grown culture was grown to a mid-logarithmic phase with an OD of 0.4–0.6 at 600 nm in LB, pelleted, washed and resuspended in 5 mM 4-(2-hydroxymethyl)-1-piperazineethanesulfonic acid (HEPES) buffer (pH 7.2) with 5 mM glucose. Equal volumes of the resulting cell suspension were added to a black 96-well plate. Subsequently, NPN was added to each well and diluted in HEPES buffer supplemented with 5 mM glucose and 5 μM carbonyl cyanide 3-chlorophenylhydrazone to attain a final concentration of 10 μM. The plate was then incubated at room temperature for 30 min in darkness. Desired concentrations of the adjuvants were subsequently added to the designated wells. The wells consisting of cells with NPN and a recognized OM permeabilizer, PMBN, served as positive control, while the wells containing only the cells and NPN served as a negative control. A SpectraMax M2 microplate reader (Molecular Devices, Union City, CA, USA) was used to measure the change in fluorescence every 30 s at an excitation and emission wavelength of 350 and 420 nm, respectively. The experiment was conducted in triplicate, and any background fluorescence was subtracted from the spectra. The plots indicate the mean ± standard deviation (SD) of the three experiments.

### 4.7. Cell Viability Assay

The cytotoxicity of the guanidinylated polymyxins was evaluated using the cell viability assay as previously described [59]. Human embryonic kidney cells (HEK293) and liver carcinoma cells (Hep G2) were cultured in flasks with Dulbecco’s Modified Eagle’s Medium (DMEM) supplemented with 10% fetal bovine serum. The cells were incubated at 5% CO_2_ in a humidified atmosphere at 37 °C. Equal volumes of media (50 μL) containing approximately 8000 cells (Hek293) or 5000 cells (HepG2) were added to wells in 96-well plates. The wells containing only media with no cells served as blanks. The plate was incubated for 24 h. Double the final desired concentrations of the agent were subsequently added to each well (experimental and blanks) in a volume of 50 μL. After 48 h incubation, PrestoBlue reagent from Invitrogen (Waltham, MA, USA,) was added to a final concentration of 10% (*v*/*v*), and plates were incubated for an additional1 h at 5% CO_2_. Fluorescence (excitation/emission, 560/590 nm) was measured with a SpectraMax M2 plate reader (Molecular Devices, United States). Values from the blank wells were subtracted from the corresponding wells with cells. The cell viability relative to the controls with a vehicle was calculated. The plots indicate the mean ± standard deviation of two experiments with five samples each. Doxorubicin, an anticancer drug, served as a positive control, and PMB served as a negative control.

## 5. Conclusions

Colistin and PMB were repurposed as effective OM permeabilizers by direct conversion to their guanidinylated analogs through a two-step synthetic route. The substitution of the primary amines of the Dab residues to guanidinium groups resulted in reduced antibacterial activity but conserved the inherent ability of polymyxins to permeabilize the OM comparable to PMBN. Utilized as adjuvants, GCol and GPMB synergized with various antibiotic classes, particularly improving the activity of antibiotics such as rifampicin, erythromycin, ceftazidime and aztreonam. In the case of rifampicin and erythromycin, the MICs in combination with the guanidinylated polymyxins were reduced below the susceptibility breakpoint against several MDR clinical isolates, indicating that these adjuvant/antibiotic combinations restored susceptibility in previously resistant isolates. On the other hand, the potency of ceftazidime and aztreonam were greatly enhanced with the inclusion of the guanidinylated polymyxins in a triple combination therapy with avibactam against β-lactamase harboring *P. aeruginosa*. However, preliminary studies suggest that guanidinylation may not lessen the toxicity associated with the use of polymyxins. Thus, further studies should assess the toxicity profile of these compounds in more depth. 

## Figures and Tables

**Table 1:** MICs of polymyxins and derivatives against wild-type Gram-negative bacteria.

Strain	MIC (μg/mL)				
	PMBN	GCol	GPMB	Colistin	PMB
*P. aeruginosa* PAO1	128	>128	32	1	0.5
*A. baumannii* ATCC 17978	>256	>128	32	0.5	2
*E. coli* ATCC 25922	>256	16	8	0.125	1

**Table 2:** MICs of polymyxins and derivatives against colistin-susceptible Gram-negative bacteria.

Strain	MIC (μg/mL)				
	PMBN	GCol	GPMB	Colistin	PMB
*P. aeruginosa* P259-96918	>256	32	32	0.5	≤0.0625
*P. aeruginosa* P262-101856	>256	128	16	2	0.5
*P. aeruginosa* P264-104354	>256	32	16	1	2
*A. baumannii* AB027	>256	>128	>128	0.25	0.25
*A. baumannii* AB031	>256	8	2	0.25	≤0.125
*A. baumannii* LAC-4	>256	2	0.5	0.125	0.5
*A. baumannii* 110193	>256	64	4	0.5	1
*E. coli* 107115	>256	8	8	0.125	≤0.125

**Table 3:** MICs of polymyxins and guanidinylated derivatives against colistin-resistant Gram-negative bacteria.

Strain	MIC (μg/mL)				
	PMBN	GCol	GPMB	Colistin	PMB
*P. aeruginosa* 114228	>256	>128	32	4	64
*P. aeruginosa* 101243	>256	>128	>128	1024	>128
*A. baumannii* 92247	>256	>128	128	4	8
*E. coli* 94474	>256	32	16	16	16
*E. coli* 94393	>256	16	8	4	2
*E. cloacae* 121187	>256	>128	>128	>128	>64
*E. cloacae* 118564	>256	>128	>128	>128	>64
*K. pneumoniae* 113250	>256	>128	>128	256	>64
*K. pneumoniae* 113254	>256	>128	>128	256	>64

**Table 4:** MICs of polymyxins and derivatives against β-lactamase harboring * *P. aeruginosa* clinical isolates.

Strain	MIC (μg/mL)				
	PMBN	GCol	GPMB	Colistin	PMB
PA 107092	64	16	8	0.5	0.25
PA 109084	32	4	4	0.5	0.25
PA 86052	16	2	1	1	0.25
PA 88949	16	16	4	1	0.5
PA 108590	1	2	1	1	≤0.125

* Isolates with reduced MICs by the addition of avibactam.

## Supplementary Materials

The following supporting information can be downloaded at https://www.mdpi.com/article/10.3390/antibiotics11101277/s1. Table S1: Potentiation of GCol in combination with different antibiotics against wild-type *P. aeruginosa* PAO1; Table S2: Potentiation of GPMB in combination with different antibiotics against wild-type *P. aeruginosa* PAO1; Table S3: Potentiation of GCol in combination with different antibiotics against wild-type *A. baumannii* ATCC 17978; Table S4: Potentiation of GPMB in combination with different antibiotics against wild-type *A. baumannii* ATCC 17978; Table S5: Potentiation of GCol in combination with different antibiotics against wild-type *E. coli* ATCC 25922; Table S6: Potentiation of GPMB in combination with different antibiotics against wild-type *E. coli* ATCC 25922; Table S7: Potentiation of rifampicin in combination with guanidinylated polymyxins and PMBN against XDR/MDR *P. aeruginosa* clinical isolates; Table S8: Potentiation of rifampicin in combination with guanidinylated polymyxins and PMBN against XDR/MDR *A. baumannii* clinical isolates; Table S9: Potentiation of rifampicin in combination with guanidinylated polymyxins and PMBN against XDR/MDR *Enterobacteriaceae* clinical isolates; Table S10: Potentiation of erythromycin in combination with guanidinylated polymyxins and PMBN against XDR/MDR *P. aeruginosa* clinical isolates; Table S11: Potentiation of erythromycin in combination with guanidinylated polymyxins and PMBN against XDR/MDR *A. baumannii* clinical isolates; Table S12: Potentiation of erythromycin in combination with guanidinylated polymyxins and PMBN against XDR/MDR *Enterobacteriaceae* clinical isolates; Table S13: Potentiation of ceftazidime in combination with guanidinylated polymyxins and PMBN against XDR/MDR *P. aeruginosa* clinical isolates; Table S14: Potentiation of ceftazidime in combination with guanidinylated polymyxins and PMBN against XDR/MDR *A. baumannii* clinical isolates; Table S15: Potentiation of aztreonam in combination with guanidinylated polymyxins and PMBN against XDR/MDR *P. aeruginosa* clinical isolates; Table S16: Potentiation of aztreonam in combination with guanidinylated polymyxins and PMBN against XDR/MDR *A. baumannii* clinical isolates; Table S17: Potentiation of ceftazidime and aztreonam in combination with guanidinylated polymyxins and PMBN against MDR *E. coli* 107115; Table S18: Potentiation of ceftazidime in combination with guanidinylated polymyxins and PMBN against β-lactamase harboring *P. aeruginosa*; Table S19: Potentiation of ceftazidime in combination with guanidinylated polymyxins, PMBN and 8 μM avibactam against β-lactamase harboring *P. aeruginosa*; Table S20: Potentiation of aztreonam in combination with guanidinylated polymyxins and PMBN against β-lactamase harboring *P. aeruginosa*; Table S21: Potentiation of aztreonam in combination with guanidinylated polymyxins, PMBN and 8 μM avibactam against β-lactamase harboring *P. aeruginosa*; Table S22: Susceptibility profiles of MDR/XDR *P. aeruginosa* isolates; Table S23: Susceptibility profiles of MDR/XDR *A. baumannii* isolates; Table S24: Susceptibility profiles of MDR/XDR *E. coli* isolates; Table S25: Susceptibility profiles of MDR/XDR *E. cloacae* isolates; Table S26: Susceptibility profiles of MDR/XDR *K. pneumoniae* isolates; Table S27: Susceptibility profiles of MDR/XDR *K. pneumoniae* isolates; Figure S1: Triple combination of (a) ceftazidime and (b) aztreonam with avibactam and guanidinylated polymyxins or PMBN against *P. aeruginosa* PA 109084; Figure S2: Time-kill curves of ceftazidime monotherapy, dual and triple combination with GCol and avibactam against *P. aeruginosa* PA 107092; Figure S3: Measurement of OM permeabilization via NPN uptake induced by (a) GCol and (b) GPMB with PMBN as a control against wild-type *P. aeruginosa* PAO1 cells; Figure S4: Measurement of OM permeabilization via NPN uptake induced by (a) GCol and (b) GPMB with PMBN as a control against wild-type *A. baumannii* ATCC 17978 cells; Figure S5: ^1^H NMR spectrum of GCol; Figure S6: ^13^C NMR spectrum of GCol; Figure S7: COSY NMR spectrum of GCol; Figure S8: HSQC NMR spectrum of GCol; Figure S9: HMBC NMR spectrum of GCol; Figure S10: ^13^C DEPT 135 NMR spectrum of GCol; Figure S11: ^1^H NMR spectrum of GPMB; Figure S12: ^13^C NMR spectrum of GPMB; Figure S13: COSY NMR spectrum of GPMB; Figure S14: HSQC NMR spectrum of GPMB; Figure S15: HMBC NMR spectrum of GPMB; Figure S16: ^13^C DEPT 135 NMR spectrum of GPMB; Figure S17: Mass spectrum of GCol; Figure S18: Mass spectrum of GPMB; Figure S19: HPLC chromatogram of GCol; Figure S20: HPLC chromatogram of GPMB.
