# Supplementary material for: Guanidinylated Polymyxins as Outer Membrane Permeabilizers Capable of Potentiating Rifampicin, Erythromycin, Ceftazidime and Aztreonam against Gram-Negative Bacteria
Source: Antibiotics (Basel). 2022 Sep 20;11(10):1277. doi: 10.3390/antibiotics11101277 (PMC9598282; doi:10.3390/antibiotics11101277)
Supplement: Supplementary file 1 [file antibiotics-11-01277-s001.zip › antibiotics-1908784-supplementary.pdf]

Supplementary Material

Contents

Synergistic studies of guanidinylated polymyxins with different antibiotics against wild-type Gram-negative bacteria 2

Synergistic studies of guanidinylated polymyxins with rifampicin, erythromycin, ceftazidime and aztreonam against multidrug-resistant Gram-negative bacteria 7

Triple combination studies of guanidinylated polymyxins and PMBN with ceftazidime or aztreonam and avibactam against β-lactamase harboring *P. aeruginosa* 15

Time-kill curves 17

Outer membrane permeabilization 18

Structure of guanidinylated polymyxins 19

NMR spectra of guanidinylated polymyxins 20

MALDI-MS spectra 26

HPLC chromatograms........ 27

Resistance phenotype of MDR/XDR clinical isolates........ 28

Synergistic studies of guanidinylated polymyxins with different antibiotics against wild-type Gram-negative bacteria

Table S1. Potentiation of GCol in combination with different antibiotics against wild-type *P. aeruginosa* PAO1

Antibiotic	MIC antibiotic (µg/mL)		MIC GCol (µg/mL)		FIC index	Absolute MIC (µg/mL) at 4 µg/mL GCol	Fold-potentiation
	Alone	Combination	Alone	Combination			
Rifampicin	16	0.125	>128	2	0.008<x<0.023	0.0625	256
Novobiocin	1024	4	>128	8	0.004<x<0.066	128	8
Vancomycin	256	8	>128	4	0.031<x<0.063	8	32
Erythromycin	256	16	>128	2	0.063<x<0.078	16	16
Clindamycin	1024	8	>128	4	0.008<x<0.039	8	128
Linezolid	1024	128	>128	4	0.125<x<0.156	128	8
Tobramycin	2	2	>128	0.25	1<x<1.002	2	1
Colistin	0.5	0.5	>128	0.25	1<x<1.002	0.5	1
Ceftazidime	2	0.125	>128	8	0.063<x<0.125	0.25	8
Aztreonam	4	0.5	>128	4	0.125<x<0.156	0.5	8
Piperacillin	4	0.5	>128	8	0.125<x<0.188	1	4
Ciprofloxacin	0.125	0.031	>128	4	0.25<x<0.281	0.03125	4
Levofloxacin	0.5	0.063	>128	4	0.125<x<0.156	0.0625	8
Moxifloxacin	1	0.063	>128	4	0.063<x<0.094	0.0625	16
Minocycline	16	0.5	>128	4	0.031<x<0.063	0.5	32
Doxycycline	8	1	>128	2	0.125<x<0.141	1	8
Chloramphenicol	32	4	>128	4	0.125<x<0.156	4	8
Pleuromutilin	1024	245	>128	4	0.25<x<0.281	256	4

Table S2. Potentiation of GPMB in combination with different antibiotics against wild-type *P. aeruginosa* PAO1

Antibiotic	MIC antibiotic (µg/mL)		MIC GPMB (µg/mL)		FIC index	Absolute MIC (µg/mL) at 4 µg/mL GPMB	Fold-potentiation
	Alone	Combination	Alone	Combination			
Rifampicin	16	0.5	>16	1	0.031<x<0.094	0.03125	512
Novobiocin	1024	2	>16	4	0.002<x<0.252	4	256
Vancomycin	256	8	>16	2	0.031<x<0.156	2	128
Erythromycin	256	4	16	2	0.141	2	128

Clindamycin	2048	8	16	4	0.254	8	256
Linezolid	1024	32	>16	4	0.031<x<0.281	32	32
Tobramycin	2	2	16	0.25	1.016	2	1
Colistin	1	1	>16	0.25	1<x<1.016	1	1
Ceftazidime	2	0.125	16	4	0.313	0.125	16
Aztreonam	2	0.25	16	4	0.375	0.25	8
Piperacillin	4	1	>16	4	0.25<x<0.5	1	4
Ciprofloxacin	0.125	0.031	>16	4	0.25<x<0.5	0.0625	2
Levofloxacin	0.25	0.031	16	4	0.375	0.03125	8
Moxifloxacin	1	0.063	16	4	0.312	0.0625	16
Minocycline	16	0.5	>16	2	0.031<x<0.156	0.5	32
Doxycycline	8	0.5	>16	2	0.063<x<0.188	0.5	16
Chloramphenicol	32	2	>16	2	0.063<x<0.188	2	16
Pleuromutilin	1024	128	>16	2	0.125<x<0.25	32	32

Table S3. Potentiation of GCol in combination with different antibiotics against wild-type *A. baumannii* ATCC 17978

Antibiotic	MIC antibiotic (μg/mL)		MIC GCol (μg/mL)		FIC index	Absolute MIC (μg/mL) at 2 μg/mL GCol	Fold-potentiation
	Alone	Combination	Alone	Combination			
Rifampicin	2	0.063	>128	0.5	0.031<x<0.035	0.0625	32
Novobiocin	8	0.5	>128	8	0.031<x<0.094	0.5	16
Vancomycin	128	64	>128	0.5	0.5<x<0.504	64	2
Erythromycin	32	32	>128	0.25	1<x<1.002	32	1
Clindamycin	1024	256	>128	8	0.25<x<0.313	512	2
Linezolid	256	128	>128	0.25	0.5<x<0.502	128	2
Tobramycin	1	1	>128	0.25	1<x<1.002	1	1
Colistin	0.25	0.25	>128	0.25	1<x<1.002	0.25	1
Ceftazidime	16	4	>128	2	0.25<x<0.266	4	4
Aztreonam	64	8	>128	16	0.125<x<0.25	64	1
Piperacillin	64	16	>128	8	0.25<x<0.313	64	1
Ciprofloxacin	1	1	>128	0.25	1<x<1.002	1	1
Levofloxacin	0.125	0.125	>128	0.25	1<x<1.002	0.125	1
Moxifloxacin	0.063	0.063	>128	0.25	1<x<1.002	0.063	1

Minocycline	0.125	0.125	>128	0.25	1<x<1.002	0.125	1
Doxycycline	0.125	0.125	>128	0.25	1<x<1.002	0.125	1
Chloramphenicol	64	64	>128	0.25	1<x<1.002	64	1
Pleuromutilin	512	32	>128	0.5	0.063<x<0.066	32	16

Table S4. Potentiation of GPMB in combination with different antibiotics against wild-type *A. baumannii* ATCC 17978

Antibiotic	MIC antibiotic (µg/mL)		MIC GPMB (µg/mL)		FIC index	Absolute MIC (µg/mL) at 2 µg/mL GPMB	Fold-potentiation
	Alone	Combination	Alone	Combination			
Rifampicin	2	0.008	16	1	0.066	0.004	512
Novobiocin	16	0.125	16	1	0.07	0.063	256
Vancomycin	128	16	16	1	0.188	16	8
Erythromycin	32	0.5	16	2	0.141	0.5	64
Clindamycin	1024	256	8	1	0.375	64	16
Linezolid	256	64	16	2	0.375	64	4
Tobramycin	2	1	8	4	1	2	1
Colistin	0.25	0.25	16	16	1.016	0.25	1
Ceftazidime	16	4	16	2	0.375	4	4
Aztreonam	32	4	16	4	0.375	32	2
Piperacillin	64	8	16	4	0.375	64	1
Ciprofloxacin	0.5	0.5	16	0.25	1.016	0.5	1
Levofloxacin	0.063	0.031	8	4	1	0.063	1
Moxifloxacin	0.063	0.008	16	8	0.625	0.063	1
Minocycline	0.25	0.063	8	2	0.5	0.063	4
Doxycycline	0.063	0.063	16	0.25	1.016	0.063	1
Chloramphenicol	64	32	16	1	0.563	32	2
Pleuromutilin	512	2	16	2	0.129	2	256

Table S5. Potentiation of GCol in combination with different antibiotics against wild-type *E. coli* ATCC 25922

Antibiotic	MIC antibiotic (µg/mL)		MIC GCol (µg/mL)		FIC index	Absolute MIC (µg/mL) at 2 µg/mL GCol	Fold-potentiation
	Alone	Combination	Alone	Combination			
Rifampicin	2	0.016	16	0.25	0.023	0.008	256

Novobiocin	128	0.5	16	0.25	0.02	0.125	1024
Vancomycin	128	16	16	2	0.25	16	8
Erythromycin	32	0.5	8	0.5	0.078	0.25	128
Clindamycin	64	4	16	1	0.125	4	16
Linezolid	128	16	8	1	0.25	16	8
Tobramycin	2	1	16	0.25	0.516	1	2
Colistin	0.125	0.125	16	0.25	1.016	0.125	1
Ceftazidime	0.25	0.063	16	1	0.313	0.063	4
Aztreonam	0.25	0.031	16	1	0.188	0.031	8
Piperacillin	2	0.125	8	1	0.188	0.125	16
Ciprofloxacin	0.008	0.008	16	0.25	1.016	0.008	1
Levofloxacin	0.016	0.008	16	0.5	0.531	0.008	2
Moxifloxacin	0.016	0.004	16	4	0.5	0.008	2
Minocycline	1	0.25	16	0.5	0.281	0.25	4
Doxycycline	1	0.25	16	0.5	0.281	0.25	4
Chloramphenicol	2	0.5	16	2	0.375	0.5	4
Pleuromutilin	64	0.25	16	1	0.066	0.25	256

Table S6. Potentiation of GPMB in combination with different antibiotics against wild-type *E. coli* ATCC 25922

Antibiotic	MIC antibiotic (µg/mL)		MIC GPMB (µg/mL)		FIC index	Absolute MIC (µg/mL) at 2 µg/mL GPMB	Fold-potentiation
	Alone	Combination	Alone	Combination			
Rifampicin	2	0.016	8	0.5	0.07	0.008	256
Novobiocin	64	0.25	8	0.5	0.012	0.25	256
Vancomycin	128	8	8	2	0.313	8	16
Erythromycin	32	0.5	8	1	0.133	0.125	256
Clindamycin	128	8	8	2	0.313	8	16
Linezolid	128	16	8	1	0.25	8	16
Tobramycin	1	1	8	0.25	1.031	1	1
Colistin	0.25	0.031	8	1	0.25	0.0313	8
Ceftazidime	0.25	0.063	8	2	0.5	0.063	4
Aztreonam	0.25	0.016	8	2	0.313	0.016	16
Piperacillin	2	0.063	8	2	0.281	0.063	32

Ciprofloxacin	0.008	0.004	8	1	0.625	0.004	2
Levofloxacin	0.063	0.031	8	0.5	0.563	0.032	2
Moxifloxacin	0.016	0.004	8	2	0.5	0.004	4
Minocycline	1	0.125	8	1	0.25	0.125	8
Doxycycline	1	0.25	8	1	0.375	0.25	4
Chloramphenicol	2	0.5	8	2	0.5	0.5	4
Pleuromutilin	64	0.25	8	0.25	0.035	0.125	512

Synergistic studies of guanidinylated polymyxins with rifampicin, erythromycin, ceftazidime and aztreonam against multidrug-resistant Gram-negative bacteria

Table S7. Potentiation of rifampicin in combination with guanidinylated polymyxins and PMBN against XDR/MDR *P. aeruginosa* clinical isolates

Stock number	MIC rifampicin (µg/mL)		Adjuvant	MIC adjuvant (µg/mL)		FIC index	Absolute MIC rifampicin (µg/mL)	Fold-potentiation	Adjuvant concentration at absolute MIC (µg/mL)
	Alone	Combination		Alone	Combination				
P259-96918	16	0.125	PMBN	>256	2	0.008<x<0.016	0.125	128	4
	16	0.031	GCol	32	4	0.127	0.031	512	4
	16	0.016	GPMB	32	4	0.126	0.016	1024	4
P262-101856	512	32	PMBN	>256	8	0.063<x<0.094	32	16	8
	512	8	GCol	64	4	0.078	4	128	8
	512	16	GPMB	32	2	0.094	2	256	8
P264-104354	16	0.25	PMBN	>256	2	0.016<x<0.023	0.25	64	2
	16	0.5	GCol	32	2	0.094	0.5	32	2
	16	0.125	GPMB	32	2	0.07	0.125	128	2
114228*	16	2	PMBN	256	8	0.125<x<0.156	2	8	8
	16	0.5	GCol	128	8	0.031<x<0.094	0.5	32	8
	16	1	GPMB	32	4	0.188	0.125	128	8
101243*	2	0.25	PMBN	>256	8	0.125<x<0.156	0.25	8	8
	4	0.063	GCol	>128	2	0.016<x<0.031	0.016	256	8
	4	0.031	GPMB	>128	2	0.008<x<0.023	0.008	512	8

*Colistin-resistant

Table S8. Potentiation of rifampicin in combination with guanidinylated polymyxins and PMBN against XDR/MDR *A. baumannii* clinical isolates

Stock number	MIC rifampicin (µg/mL)		Adjuvant	MIC adjuvant (µg/mL)		FIC index	Absolute MIC rifampicin (µg/mL)	Fold-potentiation	Adjuvant concentration at absolute MIC (µg/mL)
	Alone	Combination		Alone	Combination				
AB027	2	0.031	PMBN	>256	1	0.016<x<0.020	0.031	64	8
	1	0.004	GCol	>128	2	0.004<x<0.020	0.004	256	8
	1	0.004	GPMB	>128	1	0.004<x<0.012	0.001	1024	8
AB031	2	0.063	PMBN	>256	2	0.031<x<0.039	0.25	8	0.5

	1	0.008	GCol	8	0.5	0.07	0.008	128	0.5
	1	0.25	GPMB	2	0.5	0.5	0.25	4	0.5
110193	2	0.031	PMBN	256	4	0.016<x<0.031	0.063	32	1
	1	0.008	GCol	64	1	0.023	0.008	128	1
	1	0.008	GPMB	8	0.5	0.07	0.004	256	1
92247*	1	0.031	PMBN	256	8	0.031<x<0.063	0.031	32	8
	0.5	0.016	GCol	128	4	0.031<x<0.063	0.016	32	8
	0.5	0.016	GPMB	128	2	0.047	0.008	64	8

*Colistin-resistant

Table S9. Potentiation of rifampicin in combination with guanidinylated polymyxins and PMBN against XDR/MDR *Enterobacteriaceae* clinical isolates

Strain	MIC rifampicin (µg/mL)		Adjuvant	MIC adjuvant (µg/mL)		FIC index	Absolute MIC rifampicin (µg/mL)	Fold-potentiation	Adjuvant concentration at absolute MIC (µg/mL)
	Alone	Combination		Alone	Combination				
E. coli 107115	32	0.063	PMBN	>256	0.25	0.002<x<0.003	0.063	512	1
	32	0.063	GCol	4	0.125	0.033	0.063	512	1
	16	0.063	GPMB	4	0.125	0.035	0.063	256	1
E. coli 94393*	8	1	PMBN	>256	2	0.008<x<0.133	1	8	2
	4	0.25	GCol	16	1	0.125	0.063	64	2
	8	0.5	GPMB	8	0.5	0.125	0.031	256	2
E. coli 94474*	8	1	PMBN	>256	2	0.125<x<0.133	1	8	4
	8	0.125	GCol	32	2	0.078	0.016	512	4
	4	0.25	GPMB	16	1	0.125	0.016	256	4
E. cloacae 121187*	1	1	PMBN	>256	8	1<x<1.031	1	1	8
	1	0.25	GCol	>128	2	0.25<x<0.266	0.25	4	8
	2	0.125	GPMB	>128	8	0.063<x<0.125	0.125	16	8
E. cloacae 118564*	8	1	PMBN	>256	2	0.125<x<0.133	1	8	8
	8	0.5	GCol	>128	2	0.063<x<0.078	0.125	64	8
	8	0.125	GPMB	>128	1	0.016<x<0.023	0.063	128	8
K. pneumoniae 113250*	16	2	PMBN	>256	2	0.125<x<0.133	2	8	8
	32	0.125	GCol	>128	4	0.004<x<0.035	0.063	512	8
	32	0.125	GPMB	>128	4	0.004<x<0.035	0.063	512	8

K. pneumoniae 113254*	8	2	PMBN	>256	2	0.25<x<0.258	2	4	8
	16	0.125	GCol	>128	2	0.008<x<0.023	0.063	256	8
	16	0.25	GPMB	>128	2	0.016<x<0.031	0.063	256	8

*Colistin-resistant

Table S10. Potentiation of erythromycin in combination with guanidinylated polymyxins and PMBN against XDR/MDR *P. aeruginosa* clinical isolates

Stock number	MIC erythromycin (µg/mL)		Adjuvant	MIC adjuvant (µg/mL)		FIC index	Absolute MIC erythromycin (µg/mL)	Fold-potentiation	Adjuvant concentration at absolute MIC (µg/mL)
	Alone	Combination		Alone	Combination				
P259-96918	256	8	PMBN	>256	16	0.031<x<0.094	32	8	4
	256	8	GCol	32	8	0.281	32	8	4
	256	8	GPMB	32	4	0.156	8	32	4
P262-101856	2048	128	PMBN	>256	2	0.063<x<0.07	128	16	8
	2048	128	GCol	64	16	0.313	ND	ND	8
	2048	128	GPMB	32	8	0.313	128	16	8
P264-104354	128	8	PMBN	>256	8	0.063<x<0.094	128	1	2
	256	8	GCol	32	8	0.281	256	1	2
	256	8	GPMB	32	2	0.094	8	32	2
114228*	64	64	PMBN	256	0.125	1<x<1.0005	64	1	8
	64	32	GCol	128	16	0.5<x<0.625	64	1	8
	64	16	GPMB	32	8	0.5	16	4	8
101243*	1024	1024	PMBN	>256	0.25	1<x<1.001	1024	1	8
	1024	1024	GCol	>128	0.25	1<x<1.002	1024	1	8
	1024	512	GPMB	>128	1	0.5<x<0.508	512	2	8

*Colistin-resistant

Table S11. Potentiation of erythromycin in combination with guanidinylated polymyxins and PMBN against XDR/MDR *A. baumannii* clinical isolates

Stock number	MIC erythromycin (µg/mL)		Adjuvant	MIC adjuvant (µg/mL)		FIC index	Absolute MIC erythromycin (µg/mL)	Fold-potentiation	Adjuvant concentration at absolute MIC (µg/mL)
	Alone	Combination		Alone	Combination				
AB027	8	0.5	PMBN	256	2	0.063<x<0.07	0.5	16	8
	4	0.125	GCol	128	1	0.031<x<0.039	0.125	32	8
	4	0.125	GPMB	128	0.5	0.031<x<0.035	0.125	32	8
AB031	16	4	PMBN	>256	0.25	0.25<x<0.251	4	4	0.5
	16	1	GCol	4	0.5	0.188	1	16	0.5
	16	2	GPMB	2	0.25	0.25	0.5	32	0.5
110193	2	0.5	PMBN	>256	0.125	0.25<x<0.2504	0.5	4	1
	2	0.125	GCol	64	0.5	0.07	0.125	16	1
	4	0.25	GPMB	4	0.5	0.188	0.125	32	1
92247*	8	8	PMBN	256	0.125	1<x<1.0005	8	1	8
	8	0.5	GCol	128	8	0.063<x<0.125	0.5	16	8
	8	0.5	GPMB	128	4	0.0934	0.5	16	8

*Colistin-resistant

Table S12. Potentiation of erythromycin in combination with guanidinylated polymyxins and PMBN against XDR/MDR *Enterobacteriaceae* clinical isolates

Strain	MIC erythromycin (µg/mL)		Adjuvant	MIC adjuvant (µg/mL)		FIC index	Absolute MIC erythromycin (µg/mL)	Fold-potentiation	Adjuvant concentration at absolute MIC (µg/mL)
	Alone	Combination		Alone	Combination				
E. coli 107115	2048	16	PMBN	<256	4	0.008<x<0.023	64	32	1
	2048	16	GCol	8	0.5	0.07	4	512	1
	2048	64	GPMB	8	0.5	0.094	8	256	1
E. coli 94393*	64	2	PMBN	>256	8	0.0313<x<0.063	8	8	2
	64	8	GCol	16	2	0.25	8	8	2
	64	2	GPMB	8	2	0.282	2	32	2
E. coli 94474*	64	32	PMBN	>256	8	0.5<x<0.531	64	1	4
	64	8	GCol	32	4	0.25	8	8	4
	64	8	GPMB	16	4	0.375	8	8	4

E. cloacae 121187*	1024	1024	PMBN	256	0.25	1.001	1024	1	8
	512	512	GCol	128	0.25	1.002	512	1	8
	512	512	GPMB	128	0.25	1.002	512	1	8
E. cloacae 118564*	256	64	PMBN	>256	2	0.25<x<0.258	64	4	8
	256	64	GCol	>128	0.5	0.25<x<0.254	64	4	8
	256	32	GPMB	>128	1	0.125<x<0.133	32	8	8
K. pneumoniae 113250*	32	4	PMBN	>256	4	0.125<x<0.141	4	8	8
	64	4	GCol	>128	4	0.0625<x<0.094	4	16	8
	64	2	GPMB	>128	2	0.031<x<0.047	2	32	8
K. pneumoniae 113254*	64	16	PMBN	>256	2	0.25<x<0.258	16	4	8
	64	4	GCol	>128	4	0.063<x<0.094	4	16	8
	64	4	GPMB	>128	2	0.063<x<0.078	4	16	8

*Colistin-resistant

Table S13. Potentiation of ceftazidime in combination with guanidinylated polymyxins and PMBN against XDR/MDR *P. aeruginosa* clinical isolates

Stock number	MIC ceftazidime (µg/mL)		Adjuvant	MIC adjuvant (µg/mL)		FIC index	Absolute MIC ceftazidime (µg/mL)	Fold-potentiation	Adjuvant concentration at absolute MIC (µg/mL)
	Alone	Combination		Alone	Combination				
P259-96918	512	64	PMBN	>256	16	0.125<x<0.188	128	4	4
	512	128	GCol	32	4	0.375	128	4	4
	512	128	GPMB	32	4	0.375	128	4	4
P262-101856	8	2	PMBN	>256	8	0.25<x<0.281	2	4	8
	16	2	GCol	64	4	0.188	2	8	8
	8	2	GPMB	32	4	0.375	1	8	8
P264-104354	64	8	PMBN	>256	16	0.125<x<0.188	64	1	2
	64	8	GCol	32	4	0.25	16	4	2
	64	8	GPMB	8	2	0.375	8	8	2
114228*	4	0.5	PMBN	256	8	0.125<x<0.156	0.5	8	8
	8	0.25	GCol	128	8	0.031<x<0.094	0.25	32	8
	4	1	GPMB	16	4	0.5	0.0625	64	8
101243*	64	8	PMBN	>256	4	0.125<x<0.141	8	8	8

	64	2	GCol	>128	2	0.031<x<0.047	2	32	8
	32	4	GPMB	>128	1	0.125<x<0.133	4	8	8

*Colistin-resistant

Table S14. Potentiation of ceftazidime in combination with guanidinylated polymyxins and PMBN against XDR/MDR *A. baumannii* clinical isolates

Stock number	MIC ceftazidime (μg/mL)		Adjuvant	MIC adjuvant (μg/mL)		FIC index	Absolute MIC ceftazidime (μg/mL)	Fold-potentiation	Adjuvant concentration at absolute MIC (μg/mL)
	Alone	Combination		Alone	Combination				
AB027	2048	1024	PMBN	>256	2	0.5<x<0.508	1024	2	8
	2048	128	GCol	>128	8	0.0625<x<0.125	128	16	8
	2048	64	GPMB	>128	8	0.0313<x<0.094	64	32	8
AB031	32	32	PMBN	>256	0.125	1<x<1.0005	32	1	0.5
	32	16	GCol	8	1	0.625	32	1	0.5
	32	4	GPMB	2	1	0.625	32	1	0.5
110193	64	64	PMBN	256	0.125	1<x<1.0005	64	1	1
	64	8	GCol	64	4	0.188	ND	ND	1
	64	4	GPMB	8	1	0.188	4	16	1
92247*	16	16	PMBN	256	0.25	1<x<1.001	16	1	8
	32	8	GCol	128	16	0.25<x<0.375	16	2	8
	16	8	GPMB	128	16	0.625	8	2	8

*Colistin-resistant

Table S15. Potentiation of aztreonam in combination with guanidinylated polymyxins and PMBN against XDR/MDR *P. aeruginosa* clinical isolates

Stock number	MIC aztreonam (μg/mL)		Adjuvant	MIC adjuvant (μg/mL)		FIC index	Absolute MIC aztreonam (μg/mL)	Fold-potentiation	Adjuvant concentration at absolute MIC (μg/mL)
	Alone	Combination		Alone	Combination				
P259-96918	32	4	PMBN	>256	4	0.125<x<0.141	4	8	4
	32	4	GCol	32	16	0.625	16	2	4
	32	8	GPMB	32	4	0.375	8	4	4
	32	4	PMBN	>256	4	0.125<x<0.141	4	8	8

P262-101856	64	8	GCol	64	8	0.25	8	8	8
	32	8	GPMB	32	2	0.313	2	16	8
P264-104354	64	16	PMBN	>256	4	0.25<x<0.266	32	2	2
	64	8	GCol	32	4	0.25	16	4	2
	32	8	GPMB	8	2	0.5	8	4	2
114228*	16	8	PMBN	256	1	0.5<x<0.504	16	1	8
	16	4	GCol	128	16	0.25<x<0.375	16	1	8
	8	1	GPMB	32	16	0.625	4	2	8
101243*	32	8	PMBN	>256	1	0.25<x<0.254	16	2	8
	16	4	GCol	>128	1	0.25<x<0.258	2	8	8
	16	4	GPMB	>128	1	0.25<x<0.258	2	8	8

*Colistin-resistant

Table S16. Potentiation of aztreonam in combination with guanidinylated polymyxins and PMBN against XDR/MDR *A. baumannii* clinical isolates

Stock number	MIC aztreonam (µg/mL)		Adjuvant	MIC adjuvant (µg/mL)		FIC index	Absolute MIC aztreonam (µg/mL)	Fold potentiation	Adjuvant concentration at absolute MIC (µg/mL)
	Alone	Combination		Alone	Combination				
AB027	1024	512	PMBN	>256	2	0.5<x<0.508	512	2	8
	1024	32	GCol	>128	8	0.031<x<0.094	32	32	8
	1024	8	GPMB	>128	8	0.008<x<0.07	8	128	8
AB031	64	64	PMBN	>256	0.125	1<x<1.0005	64	1	0.5
	64	2	GCol	4	2	0.531	64	1	0.5
	64	32	GPMB	2	1	1	64	1	0.5
110193	128	128	PMBN	256	0.125	1<x<1.0005	128	1	1
	128	8	GCol	64	4	0.125	16	8	1
	1024	512	PMBN	>256	2	0.5<x<0.508	4	32	1
92247*	128	64	PMBN	256	16	0.5<x<0.563	128	1	8
	128	64	GCol	128	4	0.5<x<0.531	64	2	8
	256	32	GPMB	128	16	0.25	64	4	8

*Colistin-resistant

Table S17. Potentiation of ceftazidime and aztreonam in combination with guanidinylated polymyxins and PMBN against MDR *E. coli* 107115

Antibiotic	MIC antibiotic (μg/mL)		Adjuvant	MIC adjuvant (μg/mL)		FIC index	Absolute MIC (μg/mL)	Fold-potentiation	Adjuvant concentration at absolute MIC (μg/mL)
	Alone	Combination		Alone	Combination				
Ceftazidime	512	16	PMBN	>256	8	0.031<x<0.063	16	32	2
	512	32	GCol	8	2	0.313	8	64	2
	512	16	GPMB	8	1	0.156	8	64	2
Aztreonam	1024	32	PMBN	>256	8	0.031<x<0.063	ND	ND	2
	1024	32	GCol	8	2	0.281	32	32	2
	1024	64	GPMB	8	2	0.313	64	16	2

Triple combination studies of guanidinylated polymyxins and PMBN with ceftazidime or aztreonam and avibactam against β-lactamase harboring *P. aeruginosa*

Table S18. Potentiation of ceftazidime in combination with guanidinylated polymyxins and PMBN against β-lactamase harboring *P. aeruginosa*

Stock number	MIC ceftazidime (µg/mL)		Adjuvant	MIC adjuvant (µM)		FIC index	Absolute MIC ceftazidime (µg/mL)	Fold potentiation	Adjuvant concentration at absolute MIC (µg/mL)
	Alone	Combination		Alone	Combination				
PA 107092	64	8	PMBN	56	2	0.161	8	8	2
	64	16	GCol	8	1	0.375	8	8	2
	64	4	GPMB	4	1	0.313	4	16	1
PA 109084	64	4	PMBN	56	4	0.134	4	16	1
	128	32	GCol	8	2	0.5	32	2	1
	128	32	GPMB	4	1	0.5	16	4	1
PA 86052	64	4	PMBN	16	1	0.125	16	4	0.25
	64	32	GCol	2	0.5	0.75	64	1	0.25
	64	32	GPMB	1	0.5	1	ND	ND	0.25
PA 88949	32	4	PMBN	16	8	0.625	32	1	0.5
	32	1	GCol	8	4	0.531	32	1	0.5
	32	4	GPMB	4	2	0.625	ND	ND	0.5

Table S19. Potentiation of ceftazidime in combination with guanidinylated polymyxins, PMBN and 8 µM avibactam against β-lactamase harboring *P. aeruginosa*

Stock number	MIC ceftazidime (µg/mL)		Adjuvant	MIC adjuvant (µM)		FIC index	Absolute MIC ceftazidime (µg/mL)	Fold potentiation	Adjuvant concentration at absolute MIC (µg/mL)
	Alone	Combination		Alone	Combination				
PA 107092	16	0.5	PMBN	56	8	0.174	2	8	2
	8	1	GCol	8	1	0.25	0.5	16	2
	8	0.5	GPMB	4	1	0.313	0.5	16	1
PA 109084	16	1	PMBN	56	4	0.134	4	4	2
	16	4	GCol	8	2	0.5	4	4	2
	16	0.5	GPMB	8	2	0.281	0.5	32	2

Table S20. Potentiation of aztreonam in combination with guanidinylated polymyxins and PMBN against β-lactamase harboring *P. aeruginosa*

Stock number	MIC aztreonam (μg/mL)		Adjuvant	MIC adjuvant (μM)		FIC index	Absolute MIC aztreonam (μg/mL)	Fold potentiation	Adjuvant concentration at absolute MIC (μg/mL)
	Alone	Combination		Alone	Combination				
PA 107092	32	2	PMBN	56	8	0.206	8	4	2
	32	8	GCol	8	1	0.375	8	4	2
	64	4	GPMB	4	1	0.313	4	16	2
PA 109084	64	4	PMBN	56	0.5	0.071	4	16	1
	64	4	GCol	4	2	0.563	32	2	1
	64	16	GPMB	2	0.5	0.5	16	4	0.5
PA 86052	64	8	PMBN	16	0.5	0.156	16	4	0.25
	32	16	GCol	2	0.25	0.625	16	2	0.25
	32	16	GPMB	1	0.125	0.625	16	2	0.25
PA 88949	16	4	PMBN	16	8	0.75	16	1	0.5
	16	2	GCol	8	4	0.625	16	1	0.5
	16	2	GPMB	4	2	0.625	16	1	0.5

Table S21. Potentiation of aztreonam in combination with guanidinylated polymyxins, PMBN and 8 μM avibactam against β-lactamase harboring *P. aeruginosa*

Stock number	MIC aztreonam (μg/mL)		Adjuvant	MIC adjuvant (μM)		FIC index	Absolute MIC aztreonam (μg/mL)	Fold potentiation	Adjuvant concentration at absolute MIC (μg/mL)
	Alone	Combination		Alone	Combination				
PA 107092	16	2	PMBN	56	8	0.268	4	4	2
	8	1	GCol	8	1	0.25	1	8	2
	16	1	GPMB	8	1	0.188	1	16	1
PA 109084	8	0.5	PMBN	56	2	0.098	0.5	16	2
	8	4	GCol	4	1	0.75	4	2	0.5
	8	4	GPMB	2	0.5	0.75	4	2	0.5

a

b

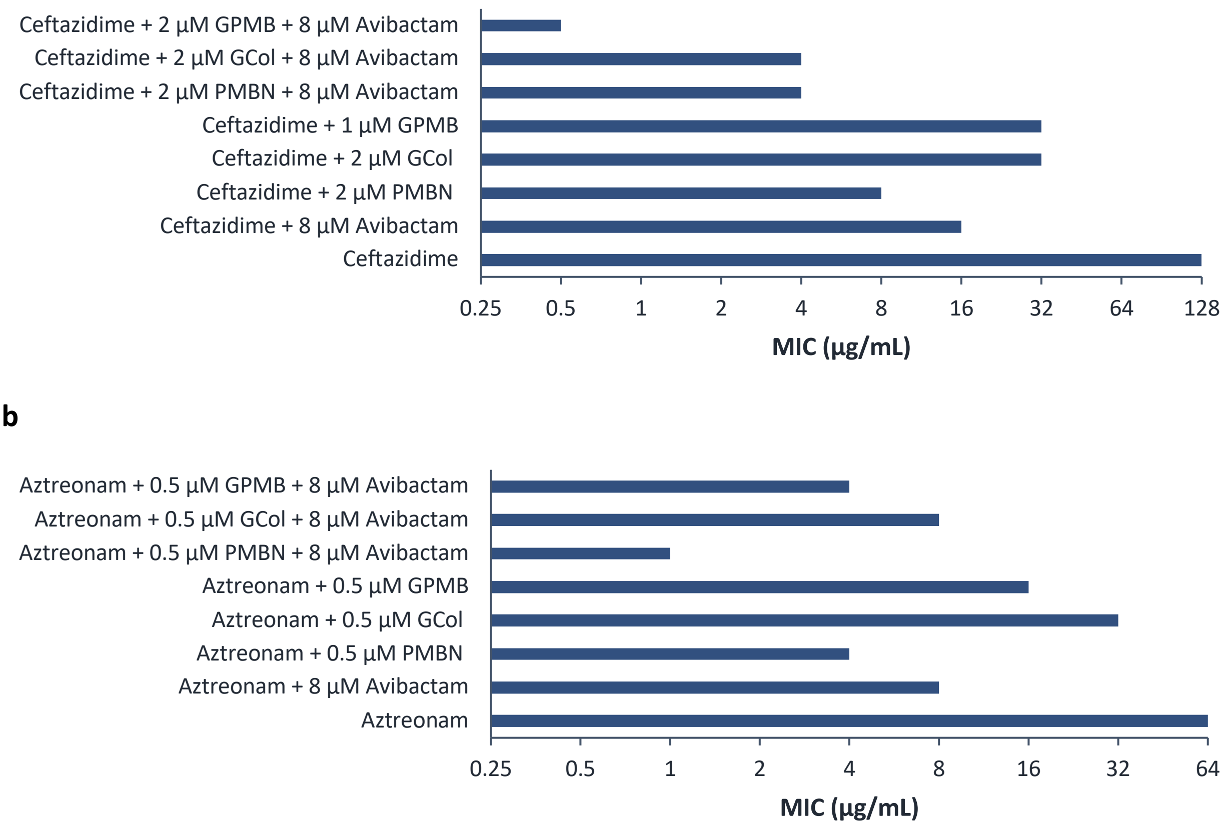

Figure S1. Triple combination of **(a)** ceftazidime and **(b)** aztreonam with avibactam and guanidinylated polymyxins or PMBN against *P. aeruginosa* PA 109084.

Time-kill curves

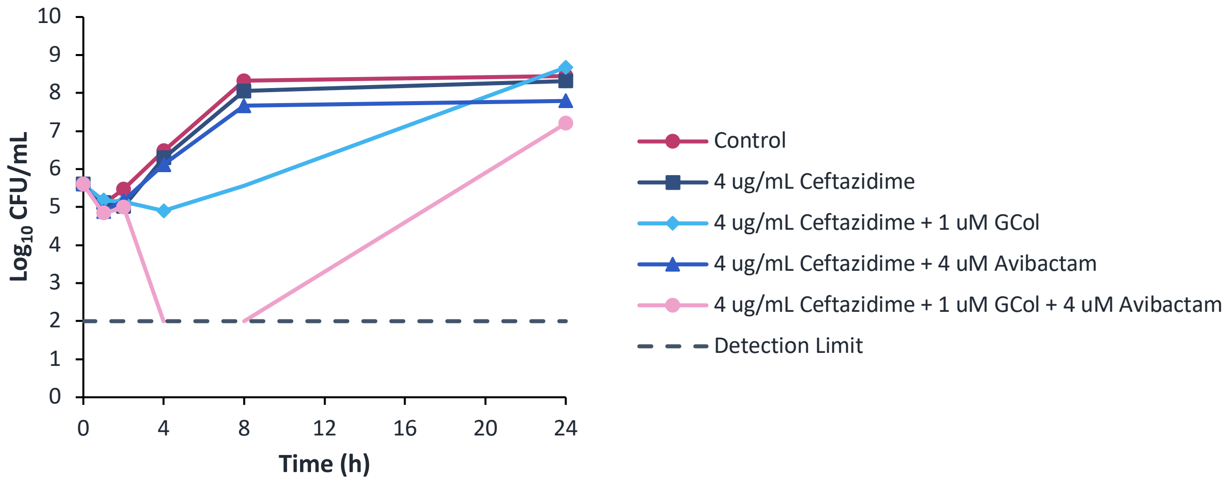

Figure S2. Time-kill curves of ceftazidime monotherapy, dual and triple combination with GCol and avibactam against *P. aeruginosa* PA 107092.

Outer membrane permeabilization

a **b**

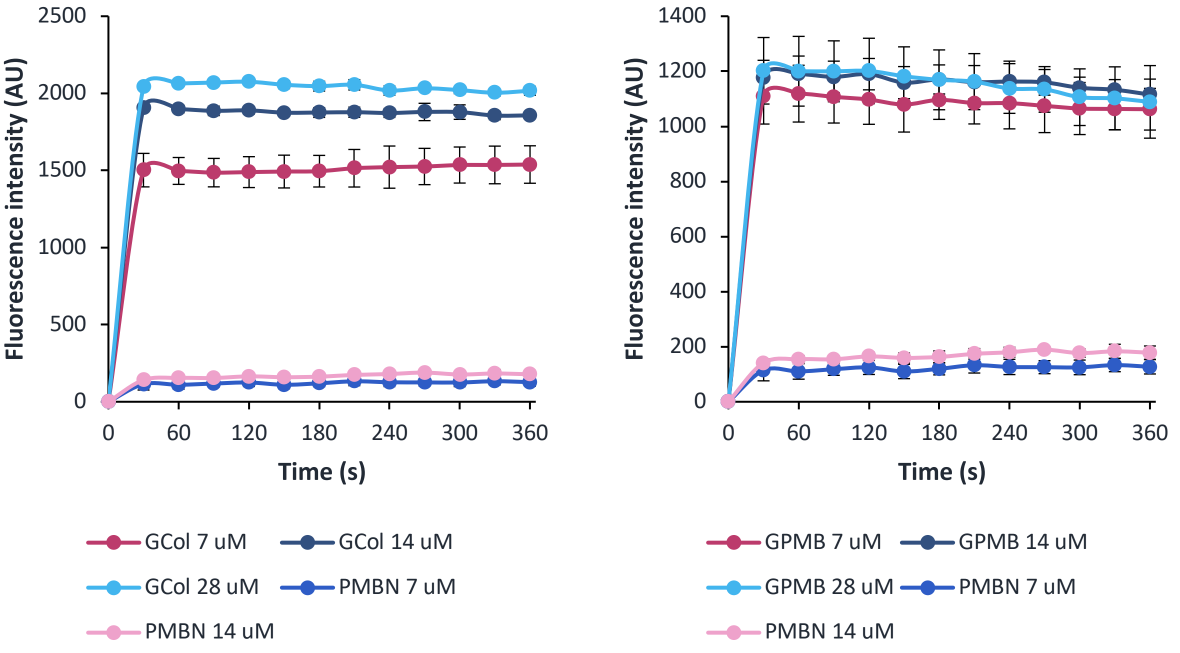

Figure S3. Measurement of OM permeabilization via NPN uptake induced by (a) GCol and (b) GPMB with PMBN as control against wild-type *P. aeruginosa* PAO1 cells.

a **b**

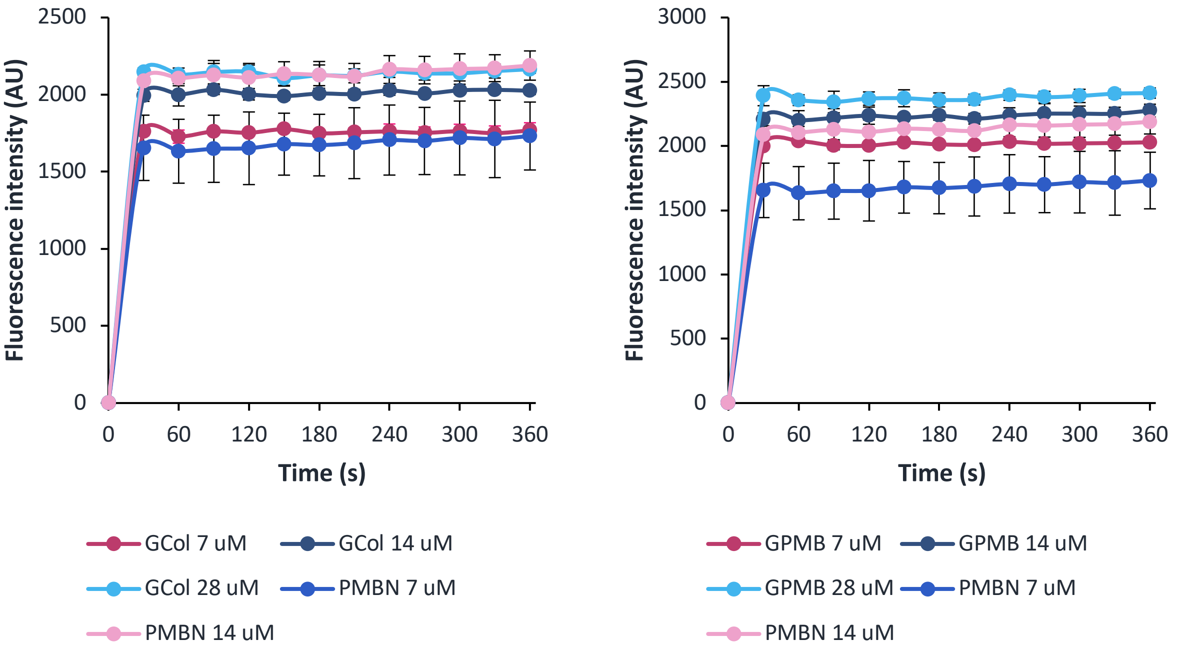

Figure S4. Measurement of OM permeabilization via NPN uptake induced by (a) GCol and (b) GPMB with PMBN as control against wild-type *A. baumannii* ATCC 17978 cells.

Structure of guanidinylated polymyxins

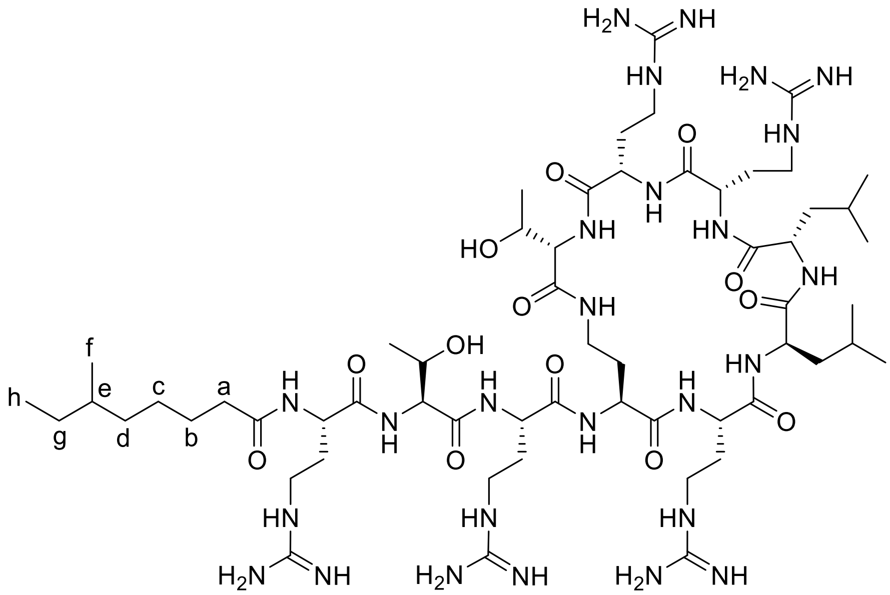

Structure of GCol A

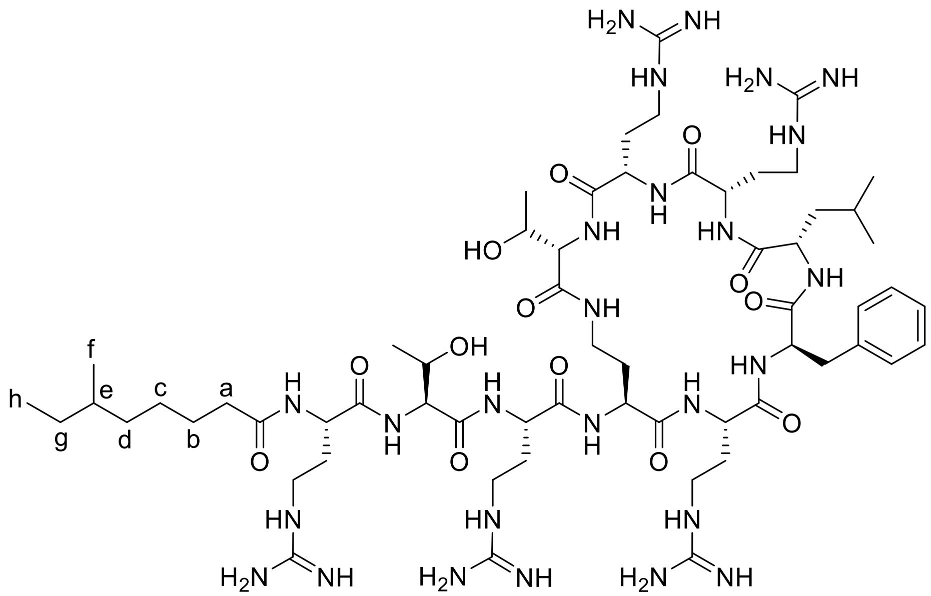

Structure of GPMB$_1$

NMR spectra of guanidinylated polymyxins

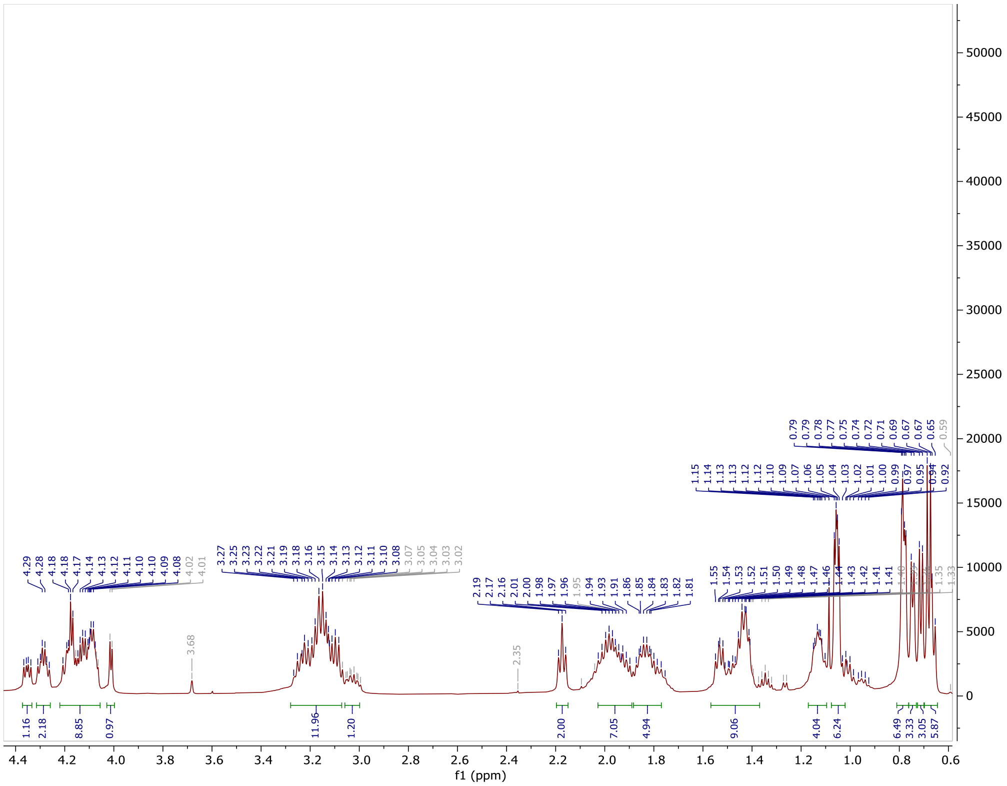

Figure S5. ^{1}H NMR spectrum of GCol.

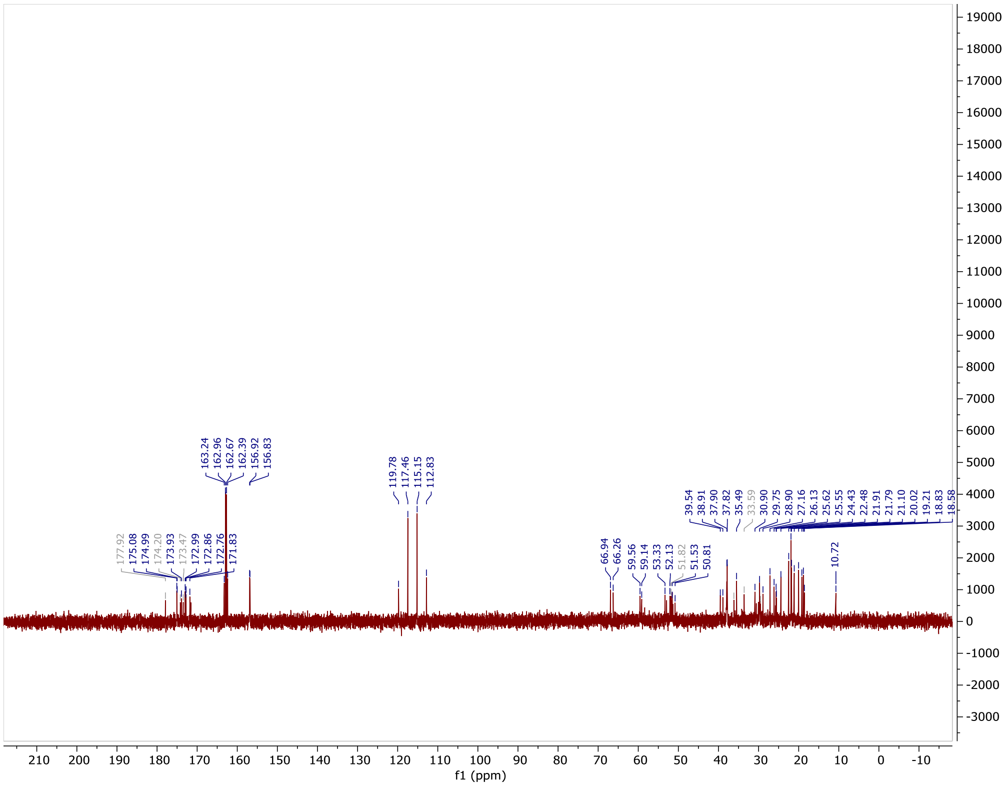

Figure S6. ^{13}C NMR spectrum of GCol.

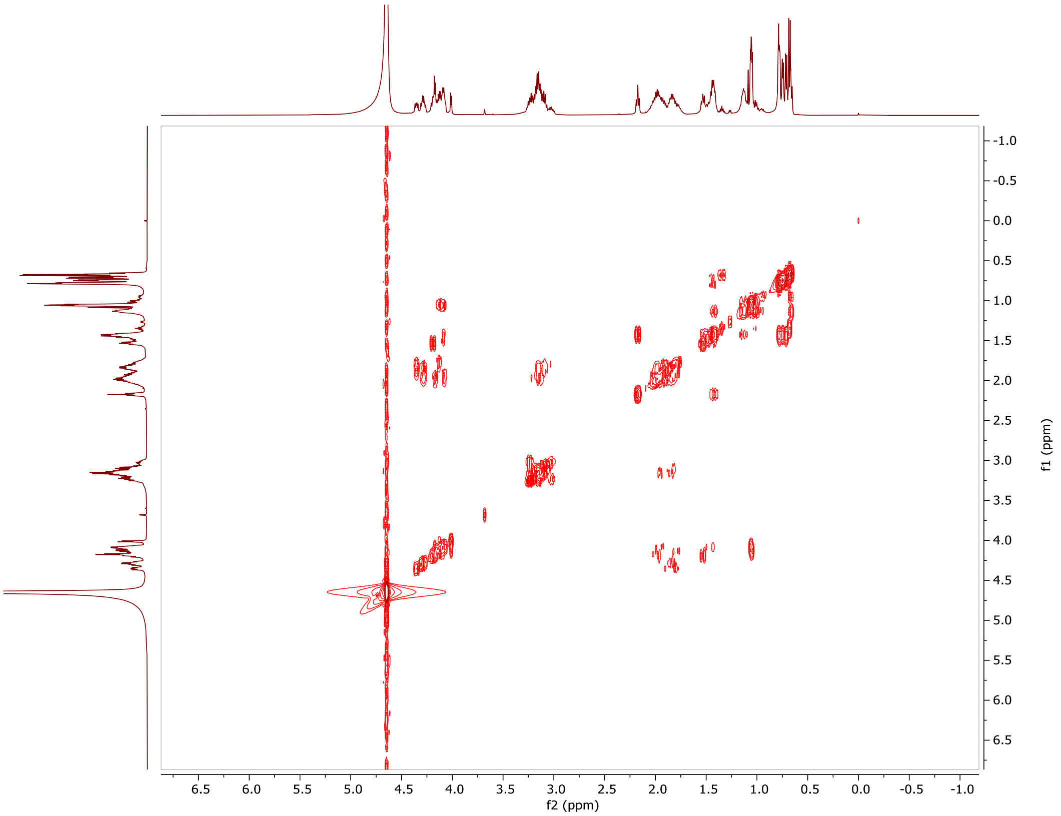

Figure S7. COSY NMR spectrum of GCol.

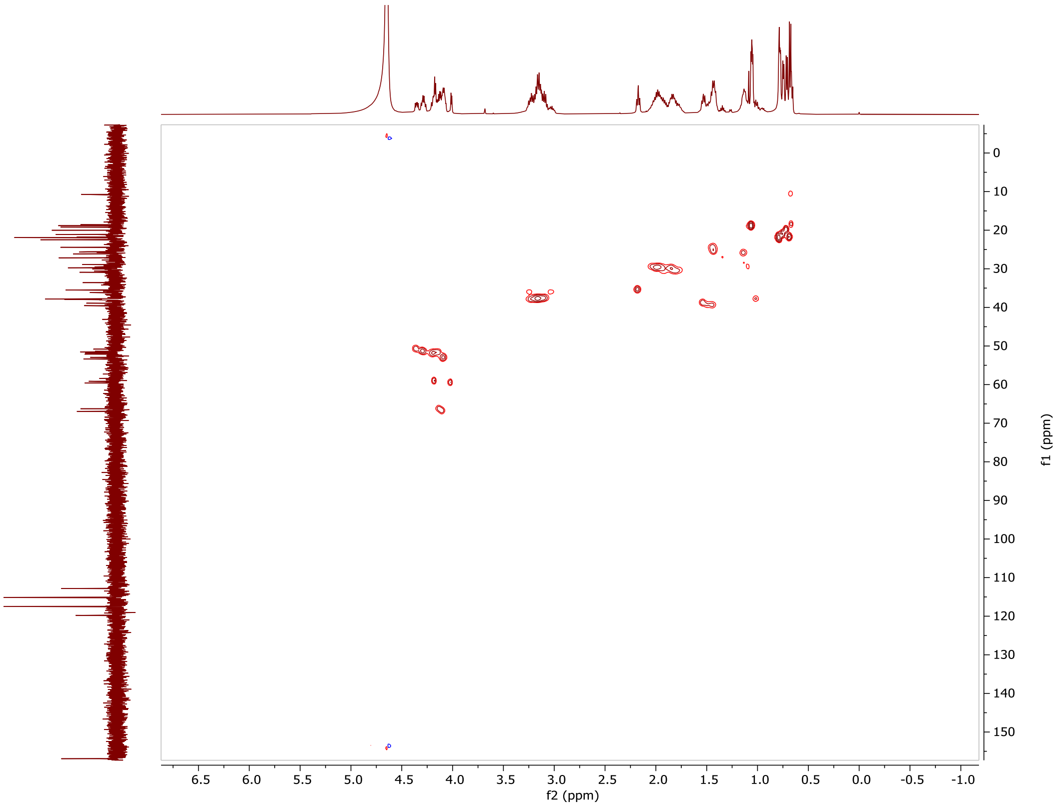

Figure S8. HSQC NMR spectrum of GCol.

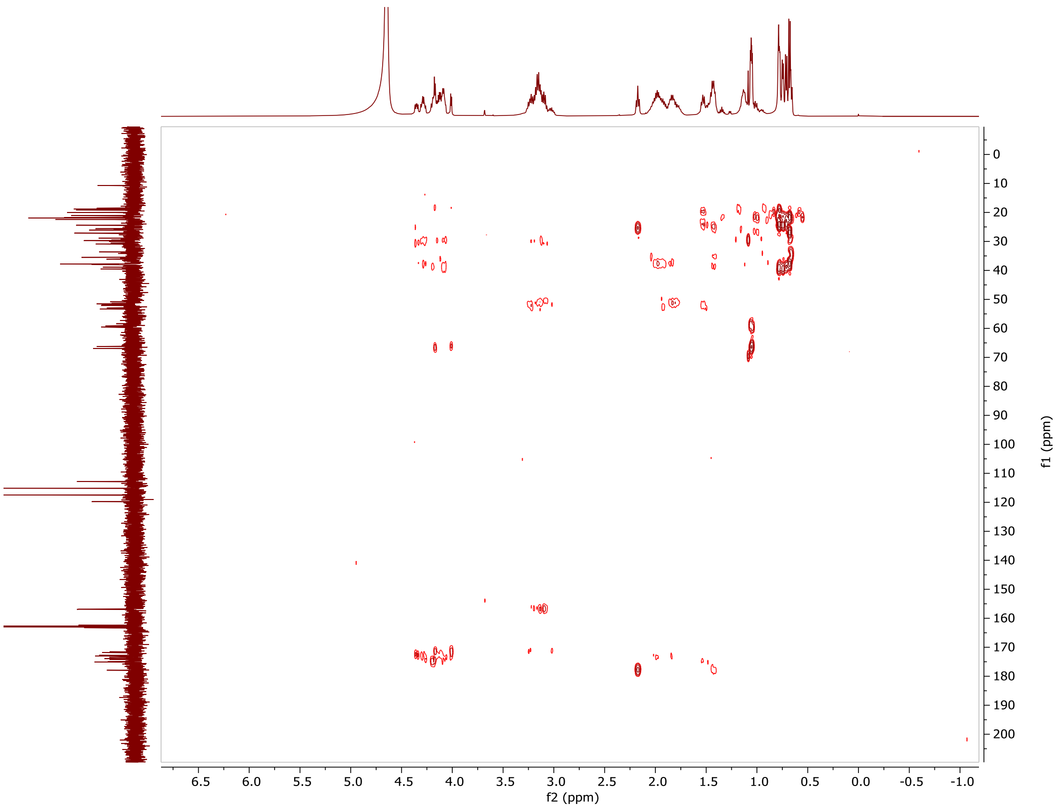

Figure S9. HMBC NMR spectrum of GCol.

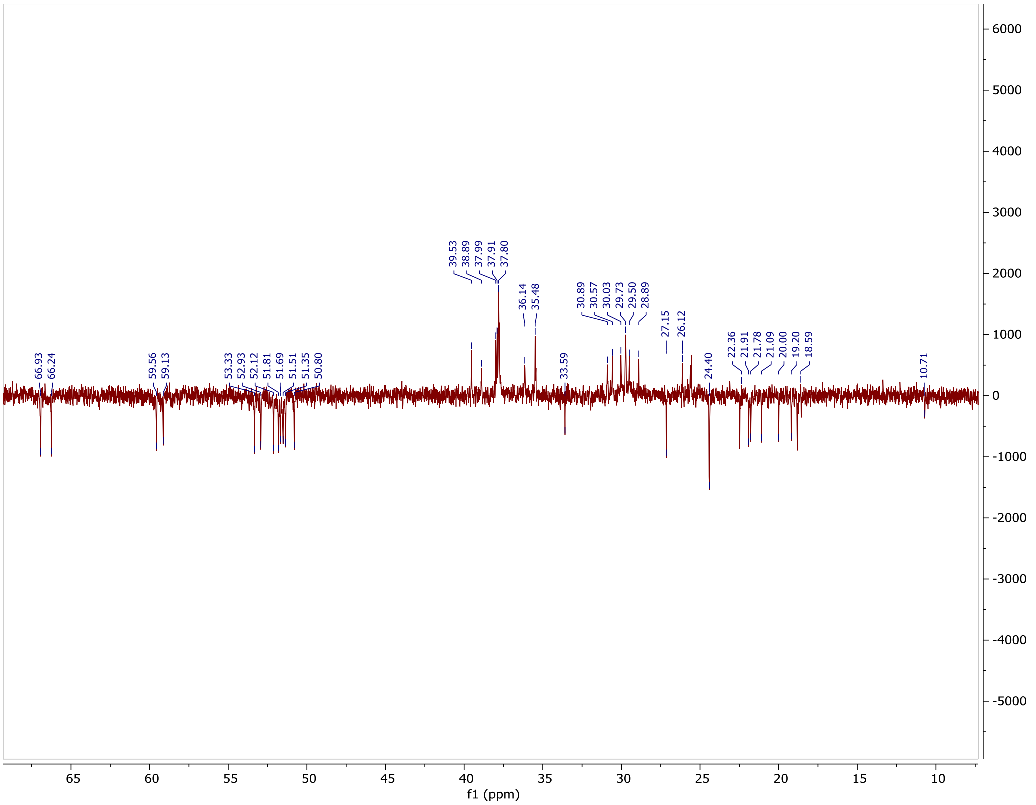

Figure S10. ^{13}C DEPT 135 NMR spectrum of GCol.

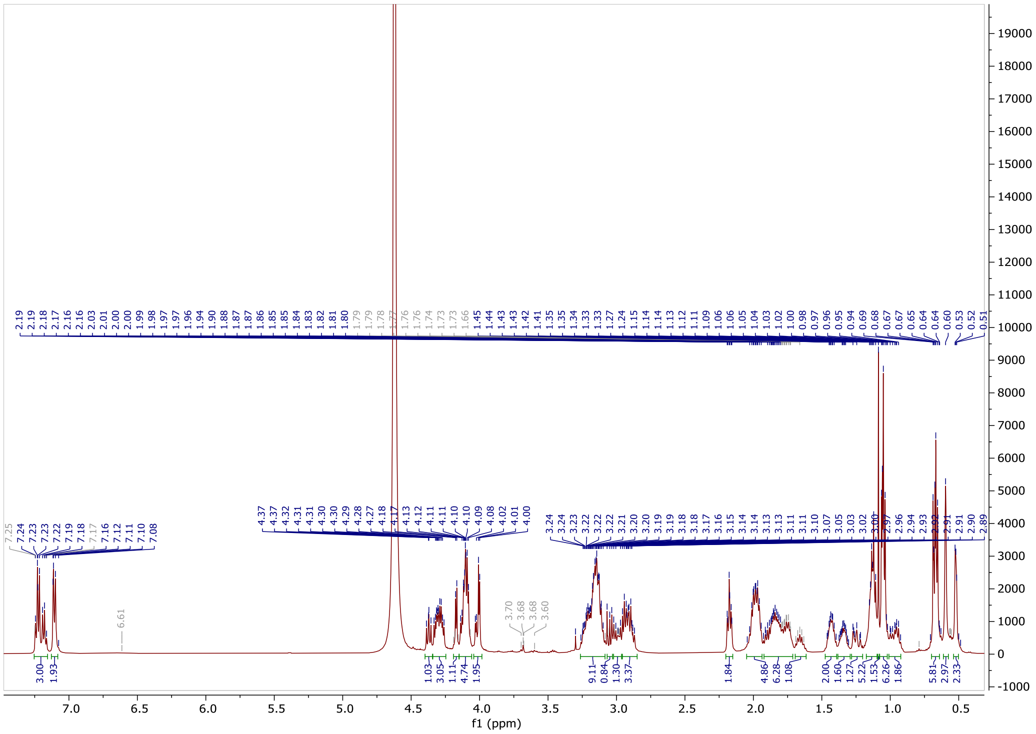

Figure S11. ^{1}H NMR spectrum of GPMB.

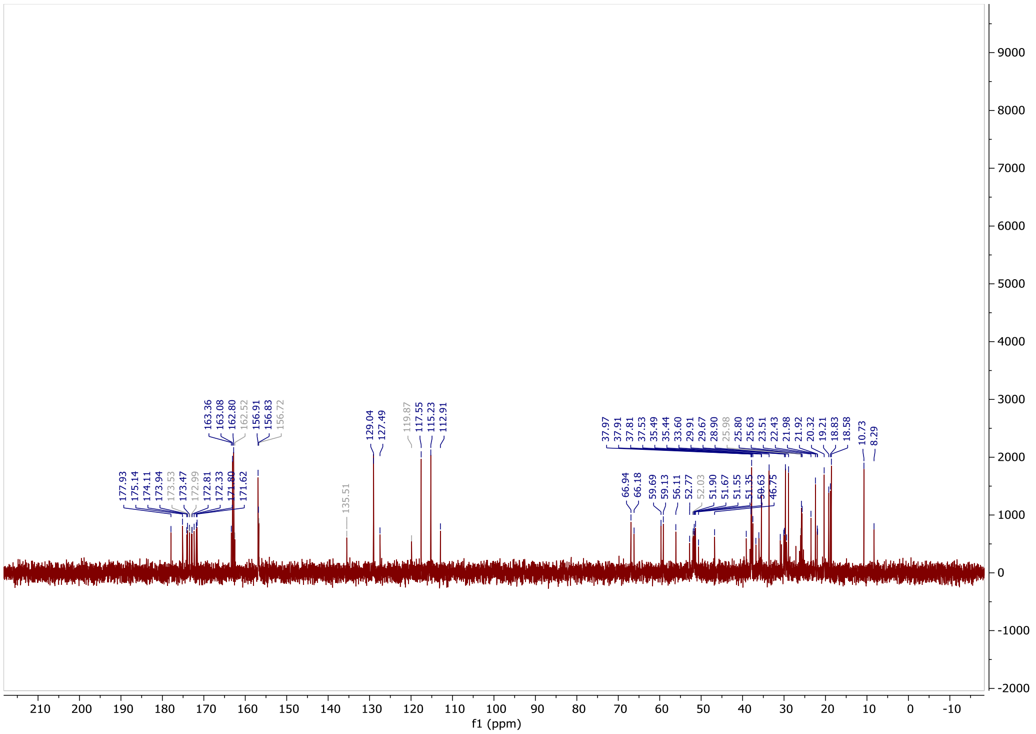

Figure S12. ^{13}C NMR spectrum of GPMB.

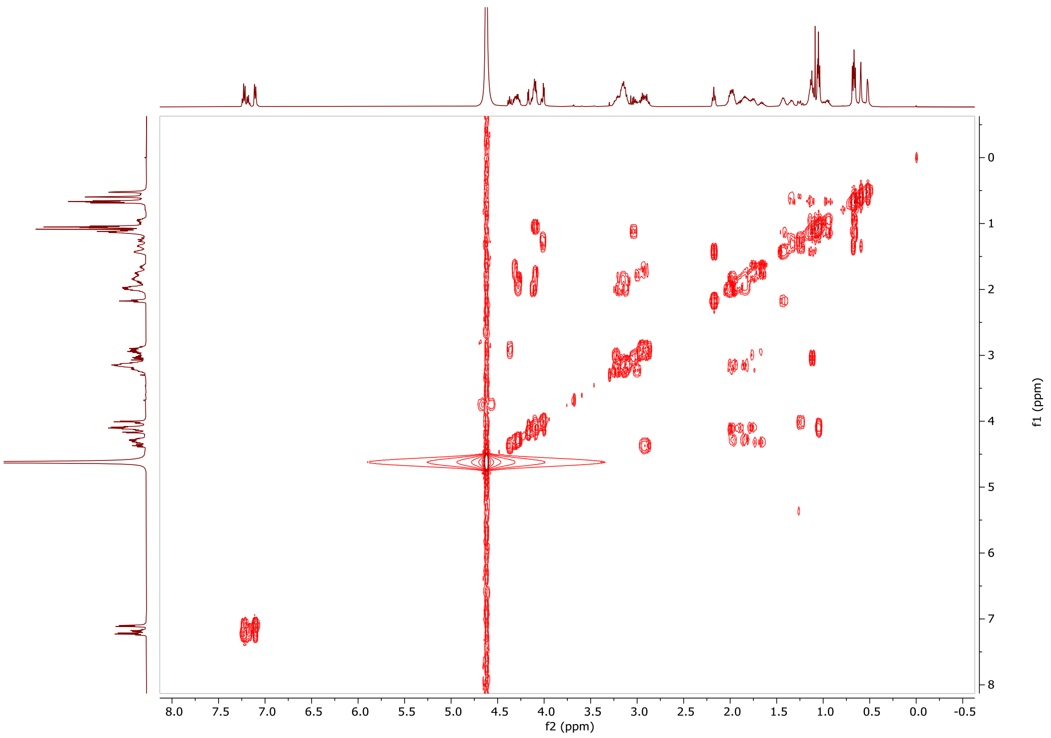

Figure S13. COSY NMR spectrum of GPMB.

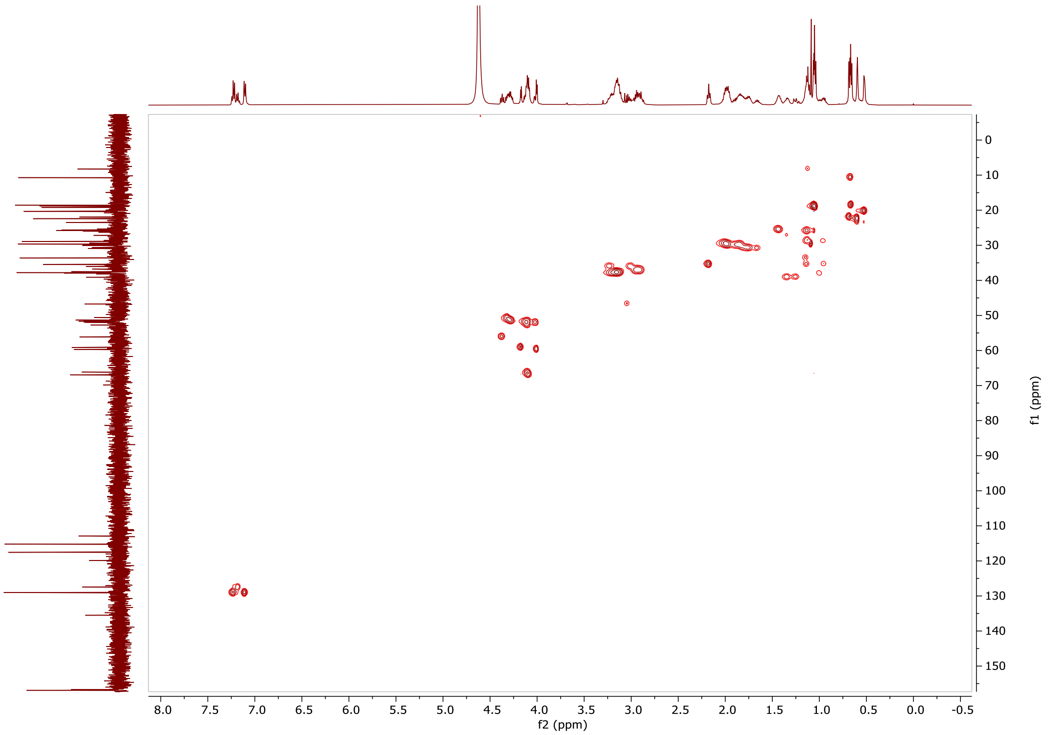

Figure S14. HSQC NMR spectrum of GPMB.

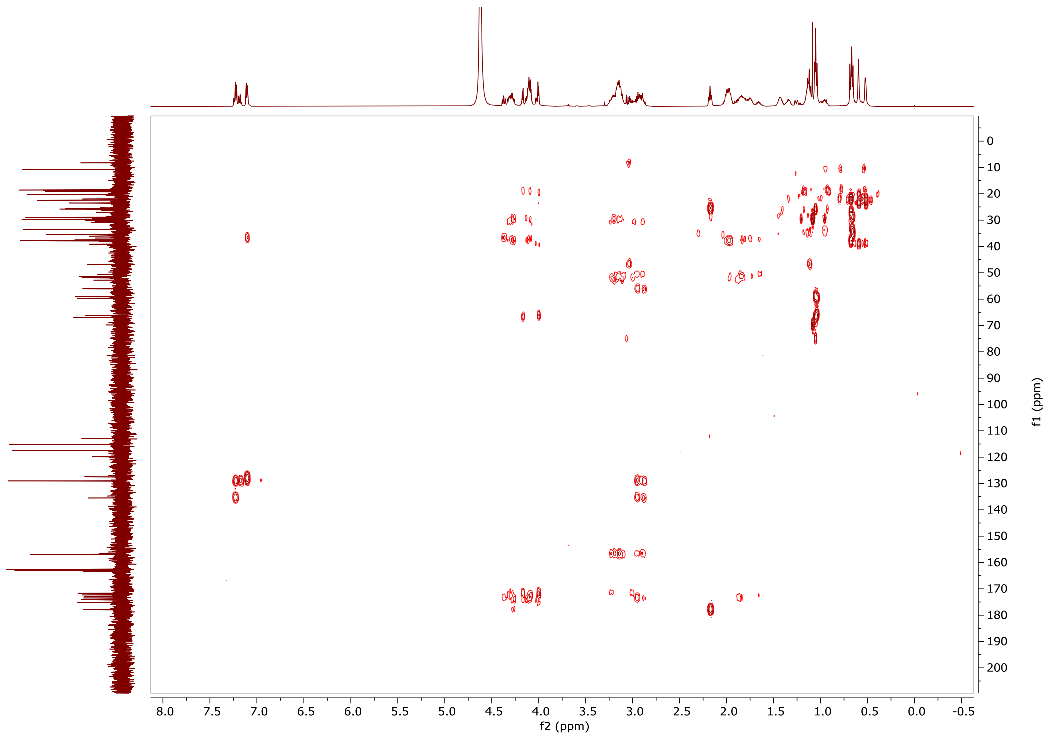

Figure S15. HMBC NMR spectrum of GPMB.

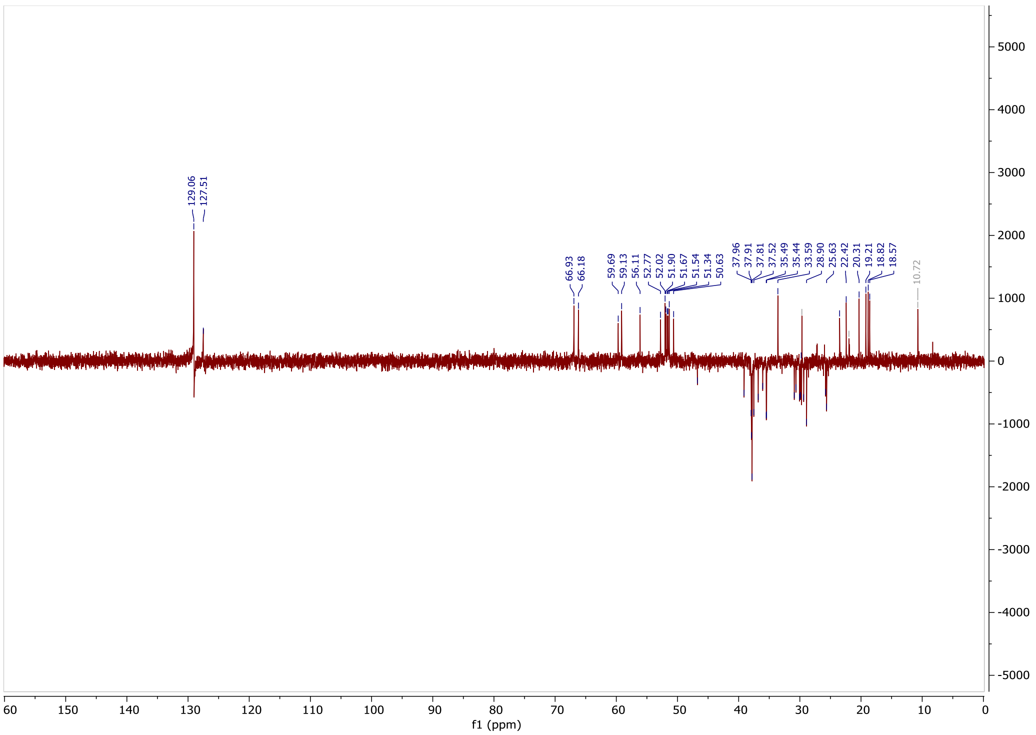

Figure S16. ^{13}C DEPT 135 NMR spectrum of GPMB.

MALDI-MS spectra

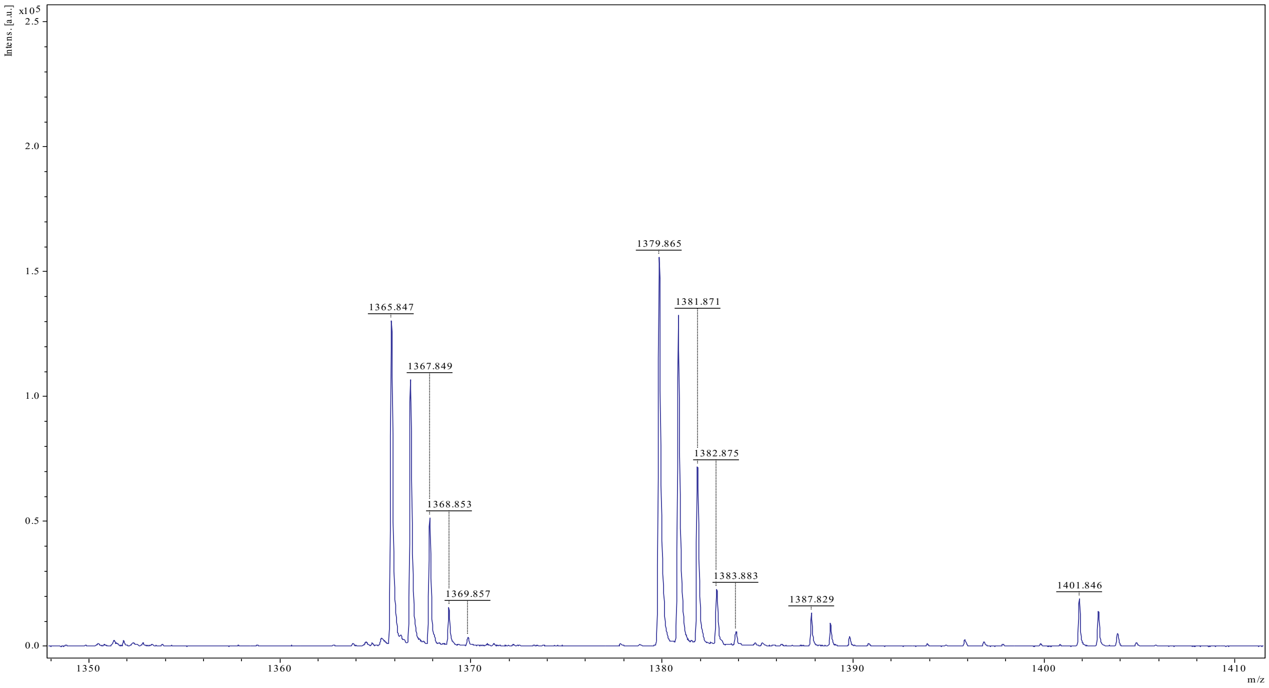

Figure S17. Mass spectrum of GCol. 1379.87 m/z and 1365.85 m/z correspond to [M+H] of GCol A and B, respectively. 1401.85 m/z and 1387.83 m/z correspond to [M+Na] of GCol A and B, respectively.

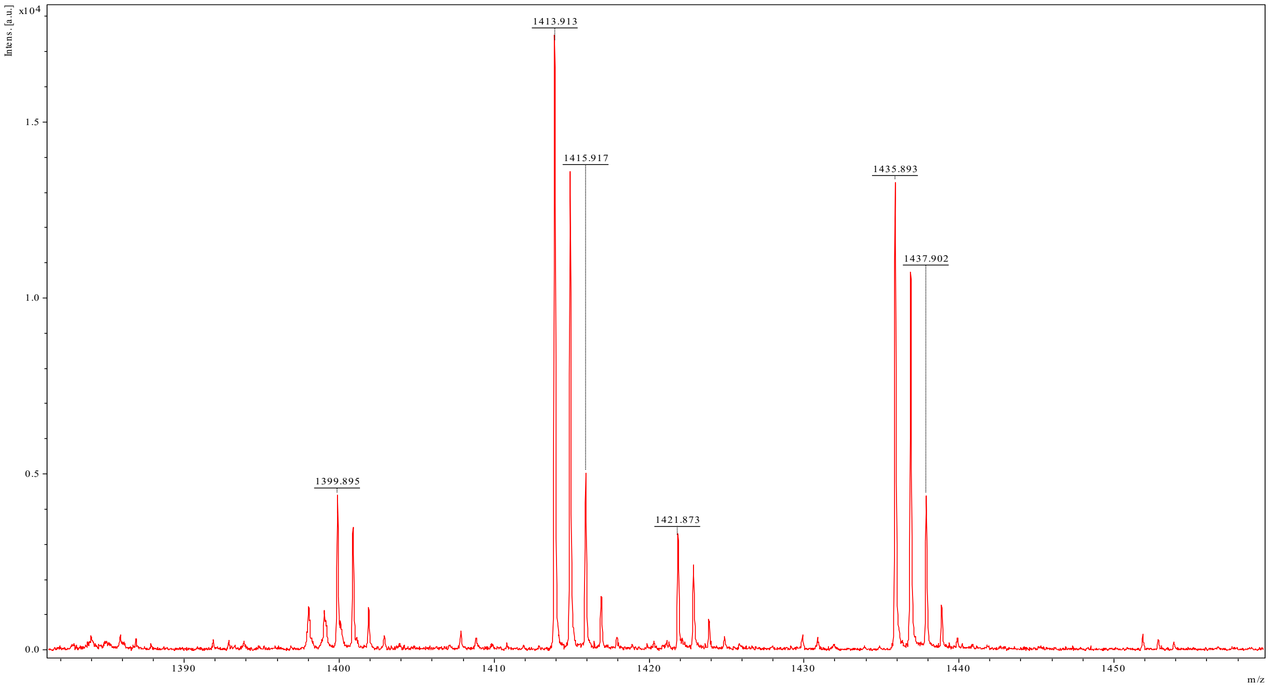

Figure S18. Mass spectrum of GPMB. 1413.87 m/z and 1399.85 m/z correspond to [M+H] of GPMB$_1$ and B$_2$, respectively. 1435.89 m/z and 1421.87 m/z correspond to [M+Na] of GMPMB$_1$ and B$_2$, respectively.

HPLC chromatograms

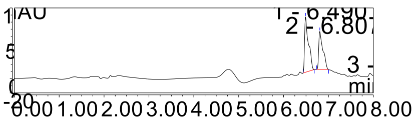

Peak number	Compound	Retention time (min)	Relative area (%)	Area (mAU*min)	Height (mAU)
1	GCol B	6.490	57.60	7.8285	78.19
2	GCol A	6.807	42.40	5.7625	53.63

Figure S19. HPLC chromatogram of GCol.

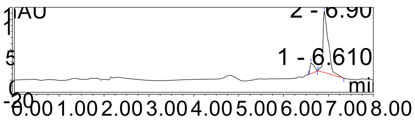

Peak number	Compound	Retention time (min)	Relative area (%)	Area (mAU*min)	Height (mAU)
1	$GPMB_2$	6.610	11.58	1.7836	18.12
2	$GPMB_1$	6.907	88.42	13.6156	101.21

Figure S20. Chromatogram of GPMB.

Eluted with 100% solvent A (20% acetonitrile with 0.1% TFA) for 5 minutes, then eluted with a linear gradient to 100% solvent B (50% acetonitrile and 20% methanol with 0.1% TFA) for 5 minutes with a flow rate of 0.5 mL/min on a reverse-phase C18 column. Detection wavelength was 210 nm.

Resistance phenotype of MDR/XDR clinical isolates

Table S22. Susceptibility profiles of MDR/XDR *P. aeruginosa* isolates

Stock number	PTZ	A/C	FOX	CFZ	CTR	CPM	CTX	IMI	MER	DOR	ETP	CIP	LEV	MOX	TOB	GEN	AMK	TGC	MIN	DOX	ERC	OMC	CAM
P259-96918	64	>32	>32	>128	>64	>64	2048	32	1024	>1024	>32	>16	256	>16	256	>32	>64	32	32	32	8	64	1024
P262-101856	64	>32	>32	>128	64	32	128	32	32	16	>32	>16	64	>16	1024	>32	>64	32	64	1024	8	64	2048
P264-104354	256	>32	>32	>128	>64	32	2048	32	64	16	>32	>16	64	>16	128	>32	8	32	32	64	8	64	4096
114228	ND	ND	ND	ND	ND	ND	128	ND	8	8	ND	ND	ND	ND	2	ND	ND	ND	32	16	16	128	ND
101243	128	>32	>32	>128	>64	64	ND	16	16	16	>32	1	ND	8	128	>32	>64	ND	2	4	ND	ND	1

Table S23. Susceptibility profiles of MDR/XDR *A. baumannii* isolates

Stock number	PTZ	FOX	CFZ	CPM	CTX	C/T	IMI	MER	CIP	LEV	MOX	TOB	GEN	AMK	TGC	MIN	DOX	ERC	OMC	CAM
AB027	512	ND	>128	>128	>256	>16	32	16	>16	8	8	ND	32	>64	4	0.25	ND	0.5	1	128
AB031	4	ND	>128	4	16	>16	0.25	1	0.25	0.25	0.125	ND	<0.5	2	8	0.25	ND	0.25	2	128
110193	ND	ND	ND	ND	ND	ND	ND	ND	≤1	≤1	≤1	ND	ND	ND	ND	1	ND	ND	ND	128
92247	<1	32	128	4	ND	2	ND	4	≤0.063	ND	ND	ND	ND	<1	0.25	0.125	ND	ND	ND	ND
LAC-4	ND	ND	ND	ND	1	8	<1	<1	>4	2	ND	>4	>4	4	<4	4	<4	0.063	1	32

Table S24. Susceptibility profiles of MDR/XDR *E. coli* isolates

Stock number	PTZ	A/C	AZT	FOX	CFZ	CPM	CAZ	C/T	IMI	MER	ETP	CIP	LEV	MOX	TOB	GEN	AMK	TGC	MIN	DOX	ERC	OMC	CAM
94393	≤1	4	≤0.13	4	1	≤0.25	≤0.25	0.25	0.25	≤0.03	≤0.03	0.5	1	1	≤0.5	≤0.5	2	0.25	2	4	0.5	4	4
94474	16	>32	≤0.13	16	4	≤0.25	0.5	0.5	0.25	≤0.03	≤0.03	>16	32	16	32	16	2	1	64	32	1	16	4
107115	>512	>32	>64	>32	>128	>64	>32	>16	8	32	>32	>16	32	16	8	>32	2	0.25	32	>32	0.125	4	512

Table S25. Susceptibility profiles of MDR/XDR *E. cloacae* isolates

Stock number	PTZ	A/C	AZT	FOX	CFZ	CAZ	CPM	MER	CIP	MOX	TOB	GEN	AMK	DOX
118564	2	>32	≤0.13	>32	>128	0.5	0.25	0.13	0.06	0.13	1	1	2	4
121187	1	8	≤0.13	>32	32	0.5	0.25	0.06	0.25	1	32	>32	1	>32

Table S26. Susceptibility profiles of MDR/XDR *K. pneumoniae* isolates

Stock number	PTZ	A/C	AZT	FOX	CFZ	CPM	CAZ	C/T	IMI	MER	ETP	CIP	LEV	MOX	TOB	GEN	AMK	MIN	DOX	ERC	OMC	CAM
113250	4	4	≤0.13	1	1	1	0.5	2	0.25	≤0.03	≤0.03	≤0.06	0.13	≤0.06	≤0.5	≤0.5	≤1	2	2	1	2	4
113254	<1	2	≤0.13	1	1	1	≤0.25	0.5	0.13	≤0.03	≤0.03	≤0.06	≤0.06	≤0.06	≤0.5	≤0.51	≤	2	128	0.5	4	2

Table S27. Susceptibility profiles of β-lactamase-harboring MDR *P. aeruginosa* isolates

Stock number	PTZ	A/C	AZT	FOX	CFZ	CTR	CPM	CTP	C/T	CAZ	CAZ-AVI	IMI	MER	ETP	CIP	MOX	TOB	GEN	AMK	DOX	SXT	NFN
PA 86052	256	>32	64	>32	>128	>64	32	16	2	64	4	32	16	>32	>16	>16	1	4	16	>32	>8	>512
PA 88949	256	>32	32	>32	>128	>64	32	32	2	64	4	>32	16	32	4	8	4	16	32	>32	>8	>512
PA 107092	512	>32	64	>32	>128	>64	64	32	2	128	16	32	32	>32	>16	>16	>64	>32	8	>32	>8	>512
PA 108590	256	>32	32	>32	>128	>64	32	16	2	32	2	32	16	>32	4	16	4	16	32	>32	>8	>512
PA 109084	512	>32	64	>32	>128	>64	32	16	2	64	4	32	16	>32	4	16	4	16	32	16	4	>512

PTZ: piperacillin-tazobactam, A/C: amoxicillin-clavulanic acid, AZT: aztreonam, FOX: cefoxitin, CFZ: cefazolin, CTP: ceftobiprole, CTR: ceftriaxone, CPM: cefepime, CTX: cefotaxime, CAZ: ceftazidime, C/T: ceftolozane-tazobactam, IMI: imipenem, MER: meropenem, DOR: doripenem, ETP: ertapenem, CIP: ciprofloxacin, LEV: levofloxacin, MOX: moxifloxacin, TOB: tobramycin, GEN: gentamicin, AMK: amikacin, TGC: tigecycline, MIN: minocycline, DOX: doxycycline, ERC: eravacycline, OMC: omadacycline, CAM: chloramphenicol, CAZ-AVI: ceftazidime-avibactam, SXT: sulfamethoxazole-trimethoprim, NFN: nitrofurantoin, ND: not determined
